# Return to work after major trauma: a systematic review

**DOI:** 10.1186/s13049-025-01351-0

**Published:** 2025-03-17

**Authors:** Anne Neubert, Sebastian Hempe, Dan Bieler, Denise Schulz, Carina Jaekel, Michael Bernhard, Joachim Windolf

**Affiliations:** 1 Department of Orthopaedics and Traumatology, Medical Faculty and University Hospital Duesseldorf, Heinrich-Heine-University, Duesseldorf, Germany; 2 TraumaEvidence @ Germany Society of Trauma Surgery, Berlin, Germany; 3 Department for Trauma Surgery and Orthopedics, Reconstructive Surgery, Hand Surgery, Burn Medicine German Armed Forces Central Hospital Koblenz, Koblenz, Germany; 4 Emergency Department, Medical Faculty and University Hospital Duesseldorf, Heinrich-Heine-University, Duesseldorf, Germany

**Keywords:** Major trauma, Polytrauma, Return to work, Ability to work, Prediction, Systematic review

## Abstract

**Introduction:**

Individuals suffering from major trauma and survive, often face diverse physical, psychological, and cognitive restrictions which can influence the (health-related) quality of life and the ability to work. Even though, return to work is not necessarily related to the health status of the individual, but it is viewed as a sign of successful reintegration and is a vital parameter of recovery.

**Objective:**

The aim was to systematically review factors influencing return to work (RTW) after suffering from major trauma.

**Material and methods:**

A search on seven databases was performed. The identified publications were selected according to the inclusion criteria: adults (≥ 16 years) who suffered a major trauma (Injury Severity Score ≥ 16) in studies that explored factors associated with RTW. Risk of bias was assessed with the ‘Quality in Prognostic studies’ tool. Due to reporting quality of the included studies no meta-analysis was performed. Data were clustered, qualitatively analyzed and factors are assessed based on the strength of evidence. (PROSPERO registration: CRD42022357649).

**Results:**

12 studies with 6907 participants (mean age 45 years, 75% males, mean ISS 28) were included. The included studies had low to moderate risk of bias for most domains, the domain ‘study confounding’ had most often a high risk of bias. Many factors were identified including physical (e.g., injury locations), personal (e.g., age) but also environmental factors (e.g., preinjury income). Only four factors (age, educational level, intensive care unit (ICU) stay and Length of stay (LOS) hospital) are based on moderate or strong evidence. The identified factors reflect the complex interactions within the process of regaining the ability to work after major trauma.

**Discussion:**

This systematic review was able to map the evidence surrounding factors affecting RTW after major trauma. Most of the identified factors are currently only based on limited evidence. According to these factors, younger patients with a higher educational level who have a shorter LOS in hospital and a shorter ICU stay might have better chances of RTW.

**Supplementary Information:**

The online version contains supplementary material available at 10.1186/s13049-025-01351-0.

## Introduction

Injuries are one of the leading causes of death and disability worldwide, especially in those individuals severely injured due to a high energy trauma [1, 2]. Individuals suffering from a major trauma and survive often face diverse physical, psychological and cognitive restrictions which can influence the (health-related) quality of life and the ability to work [3]. The inability to work is a major personal, public health and financial burden. Those individuals who do not return to work (RTW) due to illness or injury experience more physical and psychological suffering. Further, individuals face reduced finances and career opportunities. This can lead to decreased self-reported health and quality of life [4, 5]. Additionally, there are high societal costs involved e.g., due to loss of productivity [6–10]. RTW is for many individuals who survived a major trauma an important goal. Even though, RTW is not necessarily related to the health status of the individual, but it is viewed as a sign of successful reintegration and is, hence, a vital parameter of recovery [8–12].

Several publications are concerned with RTW after major trauma, some of them attempt to delineate factors that might influence RTW including e.g., personal and system-related factors [13]. To date, no systematic review has been conducted that summarizes such factors in individuals after major trauma (Injury Severity Score of ≥ 16). There is a need to systematically review the existing literature regarding factors that are associated with the RTW after a major trauma. This will offer a comprehensive understanding of factors which could support the design of interventions to support individuals after major trauma. Possibly many factors are complex and have possible interdependencies beyond the trauma. The aim of this study is to systematically review the evidence regarding factors that influence RTW after a major trauma.

## Methods

This study is reported according to the Preferred Reporting Items for Systematic Reviews and Meta*-*Analyses (PRISMA) guidelines [14]. The underlying methods are based on the guides to systematic reviews and meta-analysis of prognostic studies [15, 16]. The protocol was registered on PROSPERO (CRD42022357649).

### Eligibility criteria

The eligibility criteria are shown in Table 1. After discussion in the research team, the patient age was adjusted to 16 years (18 years and older stated in protocol) as there are many adults in this age group who are already working. A major trauma is defined in this systematic review as an individual with an Injury Severity Score (ISS) of ≥ 16 or an Abbreviated Injury Scale (AIS) ≥ 3 and at least one other injury [17, 18]. In contrast to the registered protocol, studies with mixed population regarding ISS and more than 5% of patients with ISS < 16 were excluded. A higher percentage would capture a different population of those less severely injured (ISS 9-15). In addition to the protocol, studies that merely investigate the proportion of majorly injured, who returned to work without further investigation of influencing factors, were excluded.

**Table 1:** Eligibility criteria

	Inclusion criteria	Exclusion criteria
Population	• Age ≥ 16 years (working age)	• Children (age < 16 years)
	Major trauma defined as: • Injury Severity Score (ISS) of ≥ 16 • Abbreviated Injury Scale (AIS) ≥ 3 and at least one other injury	• Studies that include more than 5% of patients with ISS < 16 • Other injuries: frailty fracture, mono injuries such as isolated facial fractures, isolated closed fractures or spinal injuries, malignant disease, amputations for other reasons than due to the major trauma (e.g., diabetes mellitus), war related injuries, burns as well as psychological trauma (if not related to the major physical trauma) • Use of other score to determine major trauma which could not be translated into ISS (e.g., Hannover Score for Polytrauma Outcome, New Injury Severity Score)
Intervention	• Any intervention is eligible including but not limited to any clinical, behavioral, and multidisciplinary interventions	
Comparison	• Any comparison is eligible including	
Predictive factors	• Any factors that affect the ability to RTW	• Factors affecting other related outcomes such as disability
Outcome	• RTW or related concepts such as ability to work, time of sick leave or others	• Studies that merely investigate the proportion of those returning to work without investigation of the influencing factors
Study designs	• Any interventional and observational study with a comparison	• Editorial notes, comments, case reports/series, abstracts, books, grey literature, systematic reviews

AIS, abbreviated injury scale; ISS, injury severity score; RTW, return to work

### Search strategy

The search was performed on 09. November 2022 on several databases (MEDLINE via PubMed, CENTRAL, PEDro, TRIP, PsychINFO, Web of Science and bibnet). Additionally, the clinical trial registers, WHO ICTRP and clinicaltrials.gov, were searched. A search strategy was developed which contains the keywords polytrauma and RTW with related synonyms. The search strategy was modified to fit the syntax of each database and trial register. There were no limitations on the timeframe. A peer review of the search strategy was performed by DS. The search strategies for each database can be found in Additional file 1—Search strategy. Additionally, the bibliographies of included studies and relevant systematic reviews related to the topic were searched for potentially eligible studies. Only publications in English and German were eligible.

### Selection

Two authors (AN & SH) screened title/abstract and full text of the identified publications, independently. The selection of studies is based on the defined inclusion and exclusion criteria (Table 1). The authors used the Covidence software to screen the publications [19]. Disputes were solved in discussion.

### Data extraction

Two authors (AN & SH) extracted the data in Excel, independently. An adapted version of the data extraction sheet by the Cochrane Methods Prognosis Group was used guided by the data extractions items described in Moons (2014) [16, 20]. The data extraction sheet was tested on two studies and adjusted accordingly. Disputes between the two authors were solved in discussion. Data on study characteristics (e.g., study design, setting), patient-related data (e.g., demographic data, comorbidities), trauma-related data (e.g., ISS, mechanism of injury, organ involvement, brain/head injuries), work related data (e.g., duration of sick leave), as well as factors affecting RTW (including statistical methods used) investigated by the included studies were extracted.

### Risk of bias

For the assessment of risk of bias and the sufficiency of reporting, the Quality in Prognosis Studies (QUIPS) Tool was used as recommended by Cochrane . The QUIPS tool relies on six domains, 1) study participation, 2) attrition, 3) prognostic factor measurement, 4) outcome measurement, 5) study confounding and 6) statistical analysis and reporting [21, 22]. The tool rates the RoB as well as the quality of reporting within the studies. The overall RoB was determined as shown in Table 2 [22, 23]. The sufficiency of reporting was rated as sufficient , partial , and insufficient reporting. The QUIPS assessment was carried out by two authors (AN & SH), independently. Disputes were settled by discussion.

**Table 2:** Determination of overall risk of bias

Overall rating of risk of bias	Number of domains of a total of 6 in each category		
	Low	Moderate	High
Low risk of bias	6	0	0
	4–5	1–2	0
Moderate risk of bias	3	3	0
	Any	1	1
High risk of bias	Any	≥ 2	1
	Any	Any	≥ 2
	Any	≥ 4	Any

### Synthesis

The meta-analysis was planned in the protocol to synthesize the effects of the identified factors. However, many issues appeared in the included studies that prevented a meta-analysis. Among others, the studies had missing data (e.g., statistical information about the performed analysis) and factors had different effect measurements (e.g., risk ratio, odds ratio) not comparable with each other. The studies used different measurement time points and used varying definitions for RTW and the prognostic factors. Many of these issues result in increased heterogeneity. The included studies were judged to be too heterogenous to perform a meta-analysis. Hence, a narrative analysis of the results was performed. No sensitivity analysis, subgroup analysis and analysis of publication bias were performed.

As several studies only reported factors that were found to be significant in multivariate analysis, only those factors were included in the synthesis. Factors from univariate analysis or non-regression analysis (e.g., group comparisons like the Chi-Quadrat test) were not used in the synthesis but reported in Additional file 4. If only median and interquartile range were provided by the included studies, means were calculated using the Quantile Estimation method proposed by McGrath (2020) [24].

**Fig. 1:**
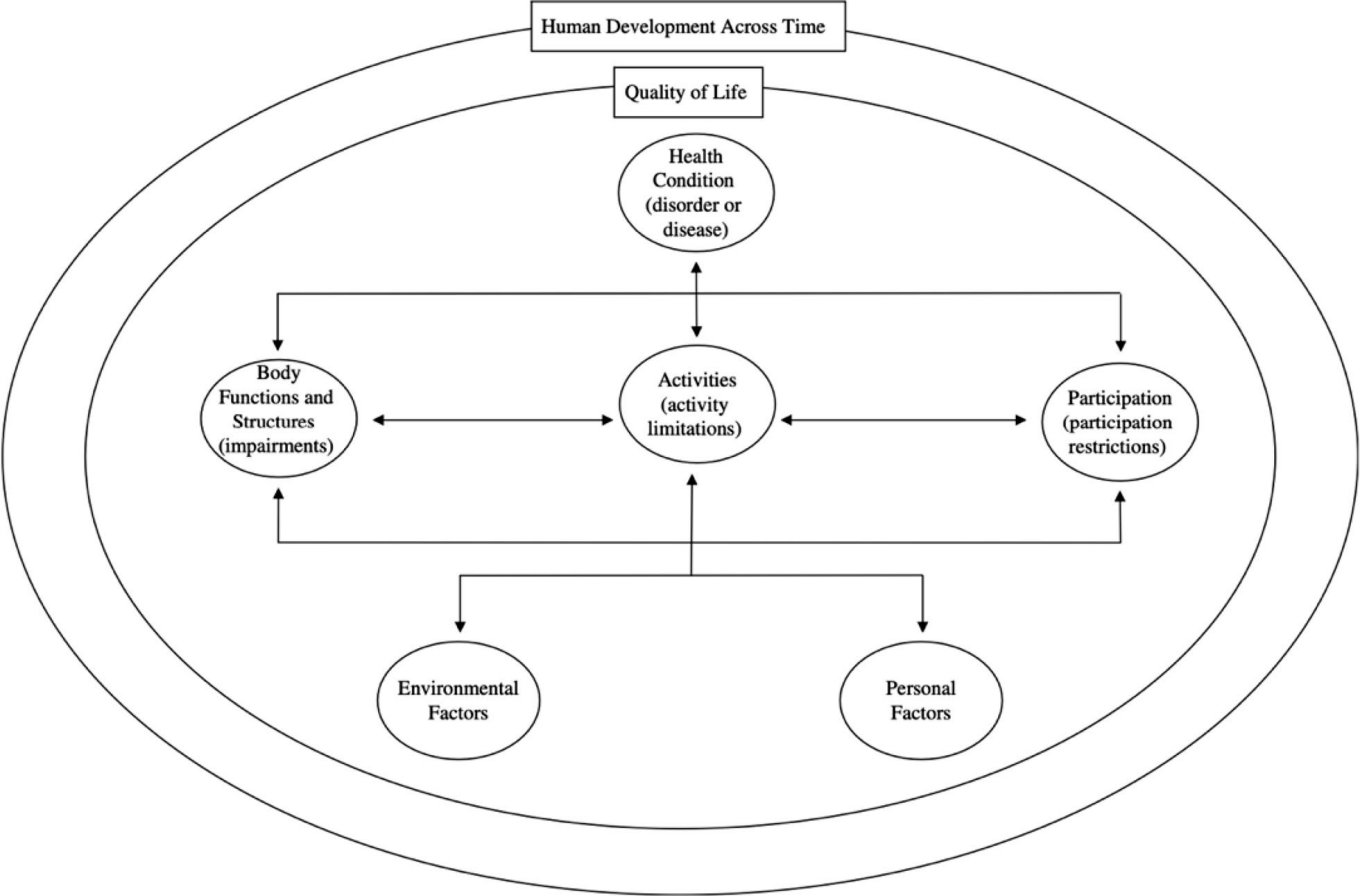
modified International Classification of Functioning, Disability and Health (ICF) framework model [25, 26]

The factors were clustered according to the International Classification of Functioning, Disability and Health (ICF) framework model. In the framework model functioning and disability are outcomes that are conditioned on the interplay between health conditions, personal, contextual, and environmental factors. Here the modified framework by McDougall (2010) is used that included also quality of life and human development across time as visualized in Fig. 1 [25]. The ICF offers a deepened understanding of the interplay between the identified factors [26].

After clustering, the strength of evidence method was used as described in several orthopedic systematic reviews on prognostic factors to synthesis the identified evidence [27–29]. The applied method of categorization of the strength of evidence is shown in Table 3. The quality of the included studies is rated based on the combined results of the RoB and sample size. Factors, that were described as having positive association with RTW in one study and as having negative association with RTW in another, are judged as inconsistent. Factors without mentioning of the direction of association are shown but not considered to contribute to the strength of evidence. To be considered as consistent evidence the effect measures and p-values should result in the same conclusion (e.g., factor X has a positive, no, or a negative association on RTW). If a factor is only reported in one study, the strength of evidence is considered limited. [27–29].

**Table 3:** Rating of strength of evidence

Strong evidence: Consistent findings in at least 2 high-quality cohort study
Moderate evidence: One high-quality cohort study and consistent findings in one or more low-quality cohort study
Limited evidence: Findings of one cohort study or consistent findings in more than one low-quality cohort study
Inconsistent evidence: Inconsistent findings irrespective of study quality

Based on the approach described in Ariëns (2000) [29]

### Results

**Fig. 2:**
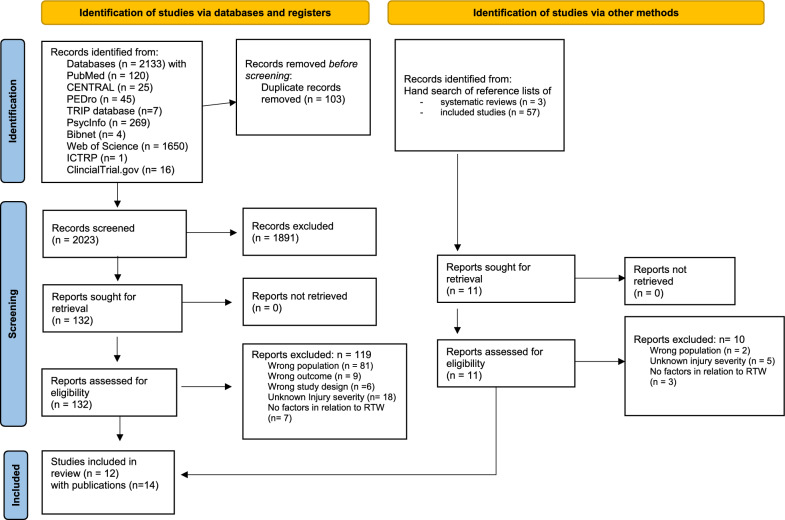
PRISMA flow chart as recommended by Page (2021) [14]

The search revealed 2,126 hits with 103 duplicates. Therefore, 2,023 titles and abstracts were screened which led to 132 full texts. Additionally due to the hand search, 60 title/abstract were screened which led to 11 full texts for the screening. The screening of full texts revealed a total of 14 publications of 12 studies that were included in this systematic review. The most common reason for exclusion of full texts was “*wrong population*” (n = 81) predominantly due to populations with an ISS mostly below 16. The selection process is illustrated in the PRISMA flowchart (Fig. 2) and an overview of the excluded studies with reasons can be found in Additional file 2.

### Population characteristics

The twelve studies included nine prospective, two retrospective and one registry-based studies. No ongoing study was identified. The included studies are from various countries like the Netherlands (n = 4) and Germany (n = 2). They were published between 1990 and 2023 as shown in Table 4. In total the systematic review includes 6,907 patients with a mean age of 45 years (mean range 31–49 years) and a mean ISS of 27.9 (mean range 21–38.9). 74.7% of the population are men. Nine studies (1,207 patients) reported the main injury mechanisms as traffic accidents (60.7%). The LOS hospital was measured in eight studies with a mean of 16.8 days in hospital (mean range 13.5–79.9 days). Five studies also measured the LOS ICU  with a mean of 22 days (mean range 15–30 days). Of the included patients 90.3% (n = 6236) were working prior to injury. Eight studies performed regression analysis for the RTW outcome [13, 30–36]. These studies developed a prediction model without external validation. Three studies only determined whether there were group differences for the outcome RTW in relation to certain characteristics. For others, it was uncertain which statistical methods were used [36–39].

**Table 4:** Characteristics of included studies

Study ID	Country of origin	Study design	Sample size	Age (Mean ± SD in years)	Sex (M/F)	ISS (Mean ± SD)
Gabbe [35]	Australia	Prospective cohort	243	35.3	199/44	30
Gross [32]	Switzerland	Prospective cohort	237	39.5 ± 20.6	180/57	27.5 ± 8.2
Grotz [39] Grotz [40]^*^	Germany	Retrospective cohort	50	33.6 ± 2.1	35/13	36.8 ± 1.6
Haas [35]	Canada	Retrospective cohort	5,341	47.3 ± 8.8	3974/1367	ø
Holtslag [13] Van Erp [41]^*^	Netherlands	Prospective cohort	214	34.8 ± 11.6	184/30	25.0 ± 11.1
Kivioja [36]	Finland	Prospective cohort	92	31	65/27	38.9 ± 1.2
Livingston [37]	USA	Existing registry	100	42	81/19	28
Post [42]	Netherlands	Prospective cohort	53	37.3 ± 13.2	43/10	23.5 ± 8.2
Simmel [31]	Germany	Prospective cohort	127	37.3 ± 11.5	66/61	35.6 ± 7.9
Soberg [30]	Norway	Prospective cohort	102	34.5 ± 13.5	84/18	28.1 ± 11.3
Van Ditshuizen [38]	Netherlands	Prospective cohort	182	49.3	116/66	21.3
Vles [34]	Netherlands	Prospective cohort	166	35	134/32	23

ISS, injury severity score; M/F, male/female; SD, standard deviation; ø, not reported

*Both publications investigate the same study population, only the results of the top publications on are used for analysis

### Return to work

All studies included determined the concept of RTW as an outcome. Additional file 3 provides an overview of definitions, measurement time points and proportions of those individuals that RTW. While in some studies patients were simply asked for their RTW status (yes / no RTW), other studies asked more detailed (full-time / part-time / change in occupation / re-training / change of working hours / retirement / unemployment / sick leave). It is unknown whether studies that measured RTW dichotomously, also included patients that RTW part-time or those that are part of a reintegration program in which they RTW on an hourly basis while still on sick leave. Additionally, some studies rated RTW only if the participants returned to a paid occupation [35, 38, 42] thereby excluding participants that are e.g. volunteers or doing care work from the analysis. Whereas, Soberg (2007) also included participants who returned to education [30, 37] and Vles (2005) considered the inability to work [35].

The median time for the outcome measurement was 3.8 years (range 6 months to 20 years). The RTW rate also varied considerably. Among the studies that only measured RTW (yes/no) it ranged from 56.5 to 79.3%. Gabbe (2008) who measured RTW six months post-injury showed a RTW rate of 58.6% [33] whereas Grotz (1997) reported it to be 64% in their cohort after a mean of 4.9 years [39]. In studies that measured the RTW more differentiated, a range of full-time RTW of 37% to 58.4% was shown. They reported a partial RTW rate between 21.5% and 65%. Further, several studies reported on unemployment/ workless rates of 7% to 20.1% and a retirement rate of 1.9% to 13% which is also reflecting the lengths of follow-up in the single studies. Similarly, the rate of change of occupation ranged from 7.6% to 29%. The difference in retirement and change of occupation rate could be a reflection of differences in health systems as well as it could be influenced by the lengths of follow up between 1 and 5.6 years, respectively. As a results of this heterogeneity, also the proportion of those RTW varied considerably between the studies.

## Reporting and risk of bias

### Reporting

Overall, the studies have a rather moderate quality of reporting, much information is missing in the publications especially in relation to prognostic factor measurement, outcome measurement, study confounding and the performed statistical analyses. Only one study, Haas (2021) reported probable confounding factors and how confounding was investigated. [35]. All other studies lack the necessary information on confounding. However, all studies showed a sufficient reporting of the study participants with adequate reporting on place of recruitment, inclusion criteria and baseline characteristics. Also, regarding study attrition most studies showed moderate or sufficient quality of reporting.

#### Risk of bias assessment

The overall RoB was assessed to be moderate to high for most studies as shown in Table 5. Several studies potentially have a bias in relation to study confounding, prognostic factor measurement, study attrition, and/or statistical analysis. Confounding was mostly not addressed at all. Further, the domain statistical analysis was rated in most studies with a moderate risk of bias. Most studies had a small sample size [30, 31, 34, 36–38, 40, 42]. Hence, probably several studies have an issue with overfitting as the sample sizes are probably too small to detect a certain effect. Kivioja (1990) and Grotz (1997) show a high risk of bias [36, 39]. Both studies did not describe any approach for prognostic factor measurement. Moreover, Kivioja (1990) have a moderate risk of bias in the areas of study participation,—attrition and statistical analysis [36].

**Table 5:**
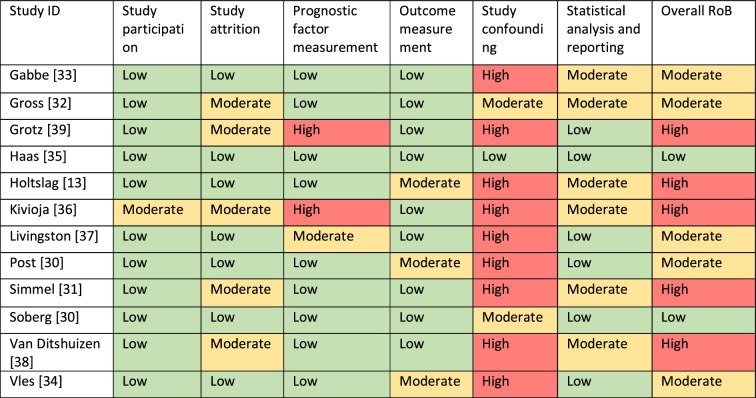
Risk of bias

*Source:* QUIPS Assessment [20]

#### Factors affecting return to work

The included studies found 32 unique factors that may influence RTW. 22 factors were only associated in single studies. All factors were clustered according to the modified ICF Framework in Table 6. It illustrates the complex interplay of personal (e.g., age), body function/structure (e.g., extremity injuries), participations and activity (e.g., physical fitness) and environmental factors (e.g., low preinjury income) combined with five factors not groupable according to ICF (e.g., ICU stay). It demonstrates, furthermore, that several factors probably have overlapping concepts e.g., educational level and low preinjury income. Additionally, it also shows that several aspects are not investigated at all or only seldom such as psychosocial, occupational or health system aspects.

**Table 6:** Grouping of factors according to ICF

Domain	Factors	Study ID
Personal factors	Age	Gabbe [33]; Haas [35]; Holtslag [13]; Kivioja [36]; Simmel [31]; Soberg [30]; Vles [34]^;^
	Sex	Haas [35]; Soberg [30];
Body function & structure	ISS	Gross [32]; Holtslag [13]; Kivioja [36]; Soberg [30]; Vles [34];
	NISS	Soberg [30]
	Extremity injury	Soberg [30]; Vles [34]
	Head injury	Haas [34]; Holtslag [13]; Kivioja [36]; Soberg [30]; Vles [34]
	Abdominal injury	Vles [34]
	Thorax injury	Vles [34]
	Spinal injury	Holtslag [13]; Soberg [30]; Vles [34]
	Number of body areas with injury	Vles [34]
	General health status	Simmel [31]
	FIM motor score	Gabbe [43]
	Head injury Symptom Checklist without anxiety	Holtslag [13]
	Co-morbidity	Holtslag [13]
Participation & activity	Physical fitness	Kivioja [36]
	Physical functioning	Soberg [30]
	Groningen Activity Restriction Scale	Holtslag [13]
	Nottingham Health Profile	Gross [32]
	Percentage of permanent impairment (AMA)	Holtslag [13]
	Educational level	Gross [32]; Soberg [30];
	Social function	Soberg [30]
	Powerful other locus of health control	Soberg[30]
Environmental factors	Low preinjury income	Haas [35]
	Time in ER	Gross [32]
	Mean nurse per day and per patient ratio	Gross [32]
	Compensable status	Gabbe [43]
	Profession	Soberg [30]
Not identifiable via ICF	ICU stay / Length of stay ICU	Haas [35]; Holtslag [13]; Simmel [31]
	LOS hospital	Haas [35]; Holtslag [13]
	Mechanical ventilation	Haas [35];
	Discharge destination	Gabbe [43]; Holtslag [13]
	Time between hospital discharge and FU	Simmel [31]

AMA, American medical association; ER, emergency room; FIM, functional independency measurement; FU, follow up; ICF, International classification of functioning, disability and health; ICU, intensive care unit; LOS, length of stay

Several of the factors are based on heterogenous definitions (ICU stay, ventilator days, spinal injury, head injury). While one study defined ICU stay as the admission to ICU [35], another defined it as an ICU stay of more than 21 days [13] and a third as the length of stay in the ICU [31]. Similarly, also the factor mechanical ventilation was defined by one study as patients that had to be mechanically ventilated [35] and by others as the length of mechanical ventilation [37, 39]. Head injury was also defined diversely (severe head injury [35], presence of any head injury [34] or head AIS [37]. Also spinal injury was defined as spinal cord injury [13] or as injury to spine and pelvis [34]. Some studies used instruments to measure the influence of certain concepts on RTW, such as using the Groningen Activity Restriction Scale to measure the concept of disability [13].

Furthermore, Additional file 4 shows all factors investigated by the included studies (including ratios and confidence intervals) including those investigated in univariate analyses but not included in multivariate analyses or assessed with other statistical analysis (e.g., Chi Square tests). These factors involve personal (e.g., profession or marital status), injury related factors (e.g., type of injury) and factors related to the post-injury functioning (e.g., functional independency measurement (FIM) score).

#### Strength of evidence

Nine factors were investigated in more than one study with the use of multivariate regression models. Table 7 shows that one factor (LOS hospital) has strong evidence whereas the factors age, educational level and ICU stay are of moderate strength of evidence. Further, sex, injury severity, head injury, extremity injury and spinal (cord) injury are of limited evidence. Sex is rated with limited evidence as the study with the largest sample size showed no association between sex and RTW . The two studies investigating spinal injuries are very heterogenous. Hence, the consistency of the evidence is questionable. Head injury is based on one high quality study, but the accompanied studies show inconsistent findings probably due to varying underlying definitions (e.g., severe head injury versus head injury). The results of injury severity as a factor are based on studies with moderate to high RoB with less than 250 participants each, but the limited evidence suggests that a lower ISS is increasing the chance of RTW. Extremity injury is based on one study with a moderate and one with a high RoB. The former states that an injury to one or more extremities is protective in relation to RTW whereas the later does not indicate the direction of association [36].

**Table 7:**
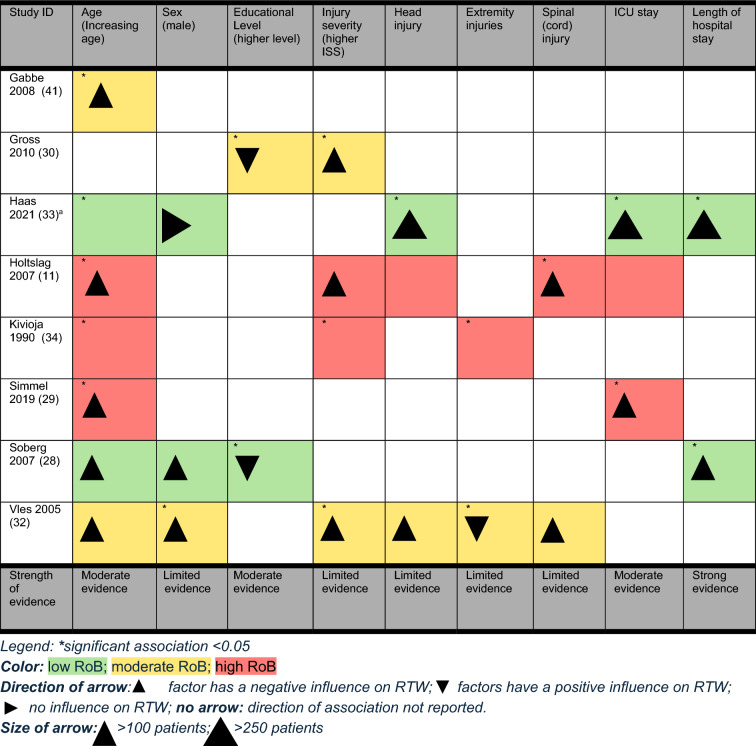
Strength of evidence rating

^*^significant association < 0.05

#### Final model of potentially influential factors

**Fig. 3:**
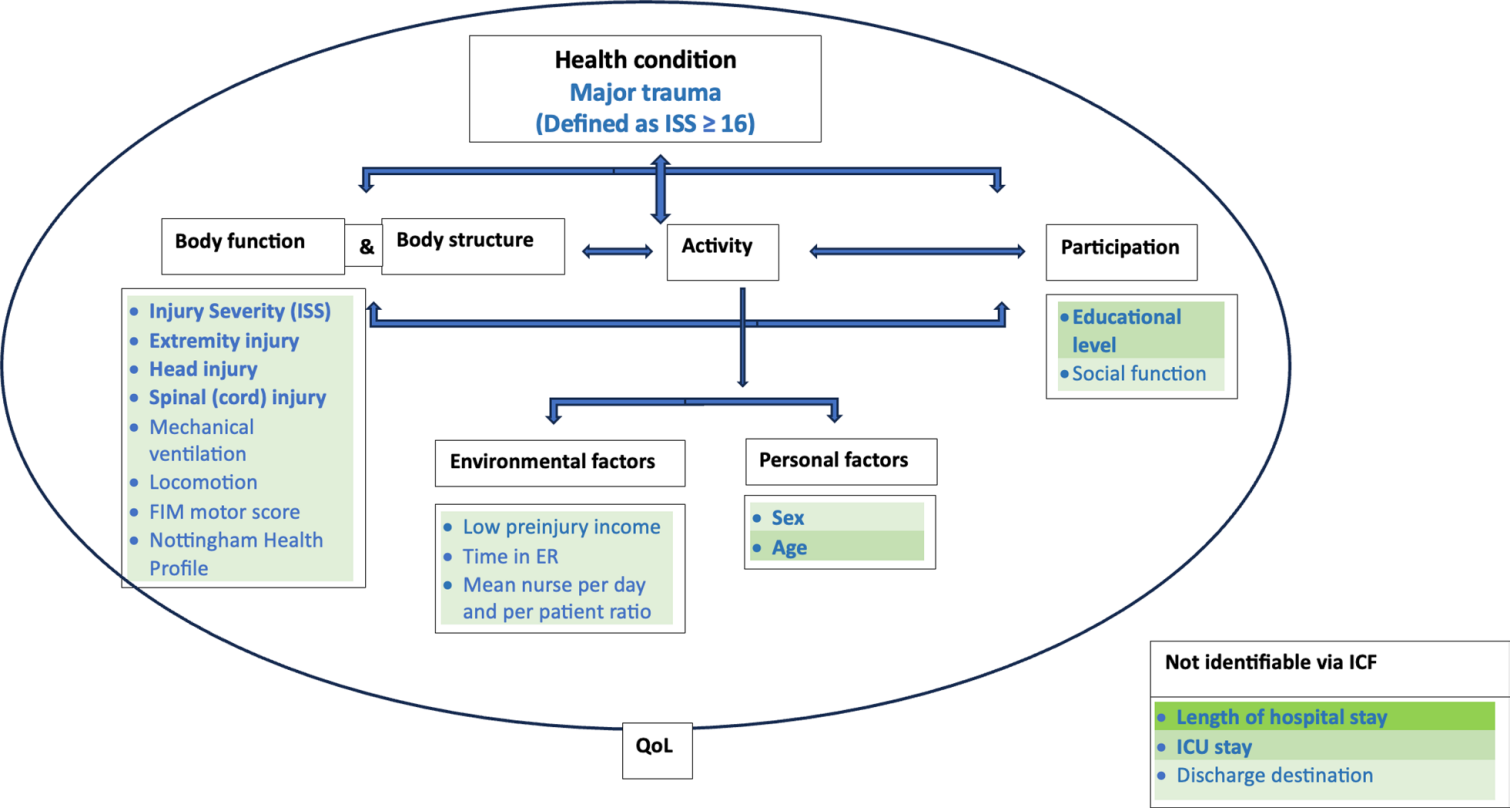
ICF for predictors of RTW after major trauma. Bold factors = factors investigated in more than 1 study

The model is based on the result of the strength of evidence rating and additional, three factors shown by one of two studies with a low RoB and sample sizes of more than 100 participants: mechanical ventilation, low preinjury income, and social functioning (Additional file 4) [30, 35]. Moreover, five factors of studies with a moderate RoB and sample sizes of more than 100 participants were shown to have a significant association with RTW: locomotion item, FIM motor score, time in emergency room (ER), mean nurse labor per day per patient and the Nottingham health profile [32, 33]. These factors have a limited strength of evidence and are integrated in the final ICF framework model of factors with a potential to influence RTW after major trauma (Fig. 3). The colors indicate the strength of evidence: the more intense the color the stronger the evidence. Several factors related to body function and structure, participation, and personal factors but also some environmental factors as well as some not integrable within the ICF model were included. The multitude of other factors shown in section “factors affecting RTW” are currently lacking the evidence base to be integrated in the final model.

## Discussion

For many severe injured patients RTW is a goal and it is certainly a determinant of functional and mental recovery after a major trauma. This systematic review was able to map the evidence surrounding factors affecting RTW after major trauma. Most of the identified factors are currently only based on limited evidence. Only four identified factors (age, educational level, ICU stay and LOS hospital) are based on moderate or strong evidence. The use of the ICF model enabled a deeper insight into the complex interactions of bodily, personal, participatory, and environmental factors in the process of regaining the capacity to RTW after major trauma. Also other studies with similar cohorts have pointed out the complex relations and that not only injury related factors but also personal, social and environmental factors account for difficulties in RTW or non-RTW [8–10, 44–49].

Factors such as ICU stay, LOS hospital or LOS rehabilitation are possibly surrogate measures for the severity of a patients’ sickness. They may reflect on the combination of the severity of injury, the general health status, and co-morbidities of the injured patient. In case of ICU stay, it could merely reflect the special need of some patients for monitoring based on their pre-existing co-morbidities. Additionally, LOS in hospital and rehabilitation is highly influenced by differences in healthcare systems as also pointed out by others [50, 51]. Even though co-morbidities were not found to be a significant factor by the included studies, others have shown its importance in regard to RTW (e.g., for psychological co-morbidity or multi-morbidity) [52, 53]. Nonetheless, these factors may just be a reflection of the short follow up period in several included studies. According to Hepp and colleagues (2011) non-RTW within the first year post-injury is mainly due to medical and rehabilitation therapy [8–10]. Gabbe und colleagues (2017) showed that 3 years post-injury still 37% had problems with mobility, 50% pain and 21% problems with self-care [54]. Hence, more sophisticated analyses of pre-injury healthy individuals compared with individuals with pre-injury co-morbidities could offer an understanding of these possible surrogate factors, a more detailed understanding of the influence of pre-injury health status on RTW.

Age as a determinant of RTW was suggest by several studies, however most of these studies also point out that this factor probably measures patients ability to recover slower also under the background of possible co-morbidities in older patients, to secure or find a job with increasing age or an incentive for early retirement [47, 48, 55]. Also, educational level was found to be associated with RTW. Herrera-Escobar and colleagues (2019) found in their cohort (average ISS 14.2) that lower educational levels have the strongest association with long-term outcomes. They also pointed out the difficulties due to the interconnectedness of concepts (educational level, income level & socio-economic status), but they showed that educational level has the strongest association of these three related concepts [56].

The influence of head or spinal injuries is likely underestimated in the present study as many studies that investigate patients with severe head injuries or spinal injuries often have a strong focus on these injured body parts and do not evaluate other body parts as influential for RTW. Further, these studies often lacked the sufficient information in relation to injury severity to be included in this systematic review [57–59].

### Strengths & limitations

The strength of this study is the systematic exploration of evidence surrounding factors that affect RTW after major trauma. This study was conducted by a multidisciplinary team on several hierarchical level which enabled a better understanding of the identified factors and their interdependencies. A broad search was performed on a range of databases which reduced a possible publication bias. Moreover, this study adhered to strict inclusion criteria which enabled the illumination of the target populations of patients with major trauma defined as an ISS ≥ 16.

However, the present analysis was restricted by several limitations. During the screening process the issue of terminology in the field of major trauma hindered the selection. The inclusion criteria had to be slightly adapted to include publications that were in line with the target population of severely injured patients with an ISS ≥ 16. Several countries define major trauma in various ways which is influenced by e.g., differences of inclusion criteria by trauma registers globally. Within the ISS group of moderate to severe injury (ISS 9–16) there are several studies that also investigate factors affecting RTW [44, 53, 55, 60–63]. However, these studies reflect on a different cohort of less severe injured and hence, had to be excluded. Nevertheless, this adaptation of inclusion criteria to have a clearly defined population may have hindered the identification of all suitable studies and may have increased the risk of evidence selection bias. When comparing our results with studies that investigate patients with an ISS ≥ 9, but mostly below ISS 16, some factors appear to be in line with our results: age, educational level, ICU admission, LOS hospital, discharge destination, ISS, extent of extremity injury [50, 55]. However, the studies also showed a wide range of other indicators e.g. sick leave prior to injury, psychiatric comorbidity [50] or alcoholism, physically demanding job, social support (esp. practical assistance), receipt of compensation (esp. workers compensation) [55]. Additionally, when the present results are compared with results from a systematic review by Clay and colleagues on RTW after acute musculoskeletal injuries several factors are in line with our findings: education (strong evidence), gender (moderate evidence), age (inconsistent evidence), injury severity (moderate evidence) [28].

Several studies used different approaches to investigate RTW. Often authors only investigate the pure fact of RTW without any differentiation (change in occupation, reduction of working hours, etc.). Many only recognize RTW if patients return to paid work which ignores those in unpaid work [32, 42, 64, 65]. Thus, it does not shade any light on those unemployed and those who lost their employment due to the injury [64, 65]. Furthermore, the included studies investigated RTW at varying measurement time points (6 months to 20 years) which is influencing the comparability of RTW rates as well as it influences the RTW rate itself. Individuals that were followed-up for 20 years could have obtained more care and could have possibly retrained in this timeframe more probable than individuals that were only follow-up for six months. Moreover, RTW rates are highly influenced by rules and regulations of social security schemes, insurances, and self-employment within countries. Countries that are in this regard more generous may have at certain measurement time points lower rates of RTW than other countries with more restrictive systems as also pointed out by Holtslag and colleagues [13]. Additionally, RTW rates are influenced by work capacity which is a somewhat different concept as the capacity reflects on the relation between occupation and the specific injury much more than the static concept of RTW. Our results show that several of the influential factors on RTW are in the domain of body function and structure and may, hence, influence also the capacity to work. A construction worker may have a longer road to achieve the work capacity needed to RTW as someone who works in a bureau. An internationally recognized definition of major trauma and RTW would help to explore determinants in more depth as heterogeneity would be reduced, leading to more valid and reliable results which improves research through better comparability and would make research projects more useful for clinical practice internationally. To develop such a definition was beyond the scope of this systematic review and would need to derive from an in-depth exploration of RTW as an outcome in major trauma research.

In relation to limitations of the included studies, all developed a prediction model for RTW after major trauma, none of the included studies validated an existing model [13, 30–39, 42]. Hence, these studies are exploratory in nature and most likely not explanatory. Most studies had a small sample size [30, 31, 34, 36–38, 40, 42] which are often more prone to high RoB– often more explorative in nature and are usually based on a convenient sample. Several studies explored many different factors for RTW which often led to spurious or even biased results. Whereas larger studies such as Haas and colleagues are more confirmatory in nature and often show better reporting and are more often protocol-driven which makes them less likely to find spurious effect estimates [15, 35]. Furthermore, in several studies the inclusion of factors in the multivariate regressions models was based on an association between each of the factors with RTW in univariate regression analyses (univariate significance testing) [7, 30–33].This approach increases the risk of predictor selection bias, especially in small samples [16]. Among others, due to the small sample sizes and probable predictor selection bias in several included studies, it is likely that the estimates of the predictive performance of the models are judged exceedingly optimistic (so-called overfitting). Consequently, the actual predictive power of the models is only poor and may be unreliable.

**Fig. 4:**
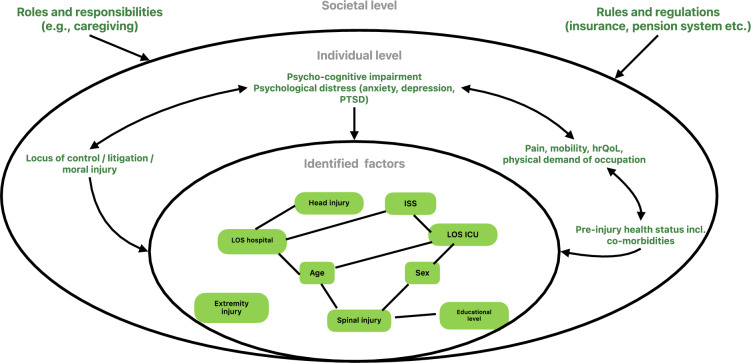
Confounder model

Only, one study addressed confounding factors. Based on the literature surrounding major trauma and the discussion on the definition of RTW and the identified factors above, a confounder model (Fig. 4) was designed﻿ to illustrate the interdependencies of the identified factors (inner circle) and other factors on individual level e.g. psychological distress [49], litigation [66, 67], and mobility (second circle) [47, 48, 66, 68], and societal level such as roles and responsibilities (surrounding layer) [69, 70]. The inner circle shows how the different factors influence each other and the outcome, for example, age is related to length of ICU and hospital stay, and the latter is concurrently linked to head injuries [71, 72]. Hence, several of the identified factors may be confounders such as age which is related to RTW but also to length of stay. Further, also the surrounding layer may serve as predictor, covariate or confounder in the interplay of RTW. Due to physical weakening and a reduced adaptability, older patients may not return to physically demanding job. The latter is again also related to education as often those with lower educational levels have physically more demanding jobs [66]. The influence of age on RTW can further be fostered by rules and regulations e.g. by incentives for early retirement in older adults [47, 48].

This model is not mutually exhaustive, possible other factors may interplay too, but it illustrates the interdependencies of factors and levels in determining the outcome RTW. This model shall serve as a basis for the exploration of interdependencies of predictors, covariates and confounders in determining RTW in major trauma survivors. It shows that there is a high need for investigation of confounders in prognostic studies in major trauma research. Hence, also the usefulness of the identified factors for research and clinical practice should be validated [73]. This study provides a comprehensive, international overview, based on which more specific research questions (e.g. definitions of RTW, confounder) could be carried out.

## Conclusion

The analysis of evidence on factors that affect RTW after major trauma showed that there are several factors that might influence RTW. Through the ICF model, it was possible to show that younger patients who have a shorter LOS in hospital might have a better chance of RTW. Similarly, those with a higher educational level and a shorter or no ICU stay might have a better chance of RTW. However, several of the identified factors, also including those with limited evidence, probably rather reflect the severity of overall sickness of the patient and therefore, it is questionable how important the single factors are in determining RTW in comparison to injury severity, co-morbidities, and general health status. Further, issues with terminology, definitions, insufficient reporting, and overfitting hampered the analysis. There is a need for more sophisticated studies of larger populations to validate these indicators and the impact for practical use such as tailored interventions for specific groups of patients after major trauma.

## Supplementary Information

Additional file 1.

Additional file 2.

Additional file 3.

Additional file 4.

## Data Availability

All data generated or analyzed during this study are included in this published article and its supplemental information files.

## Abbreviations

AIS: Abbreviated injury scale
AMA: American medical association
ER: Emergency room
ICF: International classification of function, disability and health
ICU: Intensive care unit
ISS: Injury severity score
FIM: Functional independency measurement
FU: Follow up
LOS: Length of stay
LOS hospital: Length of stay hospital
M/F: Male/female
NISS: New injury severity score
PRISMA: Preferred reporting items for systematic reviews and meta-analyses
QUIPS: Quality in prognostic studies tool
RTW: Return to work
RoB: Risk of bias
