# Supplementary material for: Return to work after major trauma: a systematic review
Source: Scand J Trauma Resusc Emerg Med. 2025 Mar 17;33:44. doi: 10.1186/s13049-025-01351-0 (PMC11917110; doi:10.1186/s13049-025-01351-0)
Supplement: Supplementary file 1 — Additional file 1. [file 13049_2025_1351_MOESM1_ESM.pdf]

Additional file 1

Search strategy

MEDLine via PubMed

1. (("Multiple Trauma"[MeSH Terms] OR "Multiple Trauma"[Title/Abstract] OR "polytrauma"[Title/Abstract] OR "major trauma"[Title/Abstract]) OR ("severe trauma"[Title/Abstract]))
2. "Return to Work"[MeSH Terms] OR "Return to Work"[Title/Abstract] OR "Returned to Work"[Title/Abstract] OR "Returning to Work"[Title/Abstract] OR "back to work"[Title/Abstract] OR "back at work"[Title/Abstract] OR "ability to work"[Title/Abstract]
3. #1 AND #2

CENTRAL

#1 MeSH descriptor: [Multiple Trauma] explode all trees
#2 "Multiple Trauma"
#3 polytrauma
#4 "major trauma"
#5 "severe trauma"
#6 #1 OR #2 OR #3 OR #4 OR #5
#7 MeSH descriptor: [Return to Work] explode all trees
#8 "Return to Work"
#9 "back to work"
#10 #7 OR #8 OR #9
#11 #6 AND #10

PEDro

1. Search: Major trauma
2. Search: Return to work AND trauma

TRIP database

Population: polytrauma
Outcome: return to work

APA PsycInfo via OVID

1 "multiple trauma".mh. or "major trauma".af. or "multiple trauma".af. or polytrauma.af. or "severe trauma".af.
2 "return* to work".mh. or "return to work".af. or "ability to work".af. or "back to work".af.
3 1 and 2

Bibnet via LIVIVO
("multiple trauma" OR polytrauma OR "major trauma" OR "severe trauma") AND ("return to work")

Web of science
(AB= (polytrauma OR major trauma OR multiple trauma OR severe trauma OR severe injury OR multiple injury)) AND AB=(return* to work OR ability to work OR back to work)

ICTRP
polytrauma OR major trauma OR multiple trauma OR severe trauma
AND
return to work OR back to work OR ability to work

Clinicaltrial.gov
Polytrauma OR major trauma OR multiple trauma OR severe trauma
Outcome measure: return to work
