# Supplementary material for: Return to work after major trauma: a systematic review
Source: Scand J Trauma Resusc Emerg Med. 2025 Mar 17;33:44. doi: 10.1186/s13049-025-01351-0 (PMC11917110; doi:10.1186/s13049-025-01351-0)
Supplement: Supplementary file 2 — Additional file 2. [file 13049_2025_1351_MOESM2_ESM.pdf]

Additional File 2

Excluded Studies

Study ID	Reason for exclusion	Reasons extended
**Abedzadeh-Kalahroudi 2015 (1)**	Wrong patient population	Mean ISS =10
**Abedzadeh-Kalahroudi 2017 (2)**	Wrong patient population	Most patients with ISS 9-15
**Ahmed 2017 (3)**	Wrong patient population	Inclusion of patients with ISS >9, no separate data for patients ISS $\geq$ 16
**Airey 2001 (4)**	No factors related to RTW	No factors related to RTW
**Anders 2013 (5)**	Wrong patient population	Mixed patient population with ISS ><16
**Anke 1997 (6)**	Wrong patient population	Includes children (age >12)
**Athanasou 2015 (7)**	Wrong patient population	ISS unclear
**Bai 2018 (8)**	Injury Severity unknown /unclear	ISS unclear
**Ballabeni 2011 (9)**	Injury Severity unknown /unclear	No ISS reported
**Baldry Currens 2000 (10)**	Wrong patient population	Includes children and ISS range 1-75
**Beck 2016 (11)**	Wrong population	Mean ISS 9 (range 5-14)
**Beck 2017 (12)**	Wrong patient population	Mean ISS = 9 (range 5-14)
**Berger-Estilita 2019 (13)**	Wrong outcome	No relation to RTW
**Brenneman 1997 (14)**	Wrong patient population	Employed patients had a mean ISS=23 ± 11.3 &
**Brenner 2011 (15)**	No factors related to RTW	No factors analyzed
**Castillo-Angeles 2021 (16)**	Wrong patient population	Median ISS = 13, (IQR: 9; 21)
**Chaboyer 2010 (17)**	Wrong patient population	ISS Range 9-14
**Collie 2019 (18)**	Wrong patient population	IQR of ISS: 14;22
**Cunha-Diniz 2022 (19)**	Wrong patient population	Most patients with ISS <16
**Czaja 2009 (20)**	Wrong patient population	Mixed population regarding ISS, no separate data for RTW for patients with ISS >16
**Daly 2022 (21)**	Wrong patient population	ISS >16
**De Munter 2020 (22)**	Wrong patient population	Mean ISS 5 (Range 4-9)
**Denu 2022 (23)**	Wrong patient population	ISS not reported, definition of population unclear
**Dinh 2016 (24)**	Wrong patient population	Median ISS= 9, IQR: 4;17
**Doan 2020 (25)**	Wrong patient population	Median ISS = 9; IQR 4-9
**Duckworth 2018 (26)**	Wrong study design	Literature review
**Faux 2015 (27)**	Wrong patient population	Most patients with an ISS <9; mean ISS 5.27
**Fleischhacker 2020 (28)**	Wrong study design	Literature review
**Folkard 2016 (29)**	Wrong patient population	Mean ISS = 12
**Fox 2013 (30)**	Wrong patient population	Mean ISS 17 ± 8
**Gabbe 2006 (31)**	Wrong patient population	ISS Range 1-66
**Gabbe 2007 (32)**	Wrong patient population	Group 1: Median ISS = 9 (Range 1-38)<br>Group 2: Median 13 (Range 1-57)
**Gabbe 2013 (33)**	Wrong patient population	More than 5% of patients with ISS <16
**Gabbe 2015 (a) (34)**	Wrong patient population	ISS ><16
**Gabbe 2015 (b) (35)**	Injury Severity unknown /unclear	ISS in this paper was dichotomized with a cutoff at ISS 12, distribution of ISS unclear
**Gabbe 2016 (36)**	Wrong patient population	Inclusion of patients ISS >12, median ISS = 17; IQR: 14;25
**Gabbe 2021 (37)**	no factors related to RTW	Only proportion of those returning to work reported
**Gabbe 2022 (38)**	no factors related to RTW	no factors related to RTW
**Giummarra 2020a (39)**	Wrong patient population	More than 30% of patients with ISS<15
**Giummarra 2020b (40)**	Wrong patient population	Includes 70% of patients with an ISS <16
**Giummarra 2021 (41)**	Wrong patient population	Includes 70% of patients with an ISS <16
**Glancy 1992 (42)**	Wrong patient population	Mean ISS 8.92
**Graham 2016 (43)**	Wrong study design	Abstract only
**Gray 2018 (44)**	Wrong patient population	Mostly non-severely/no major trauma injured patients
**Gray 2019 (45)**	Injury Severity unknown /unclear	ISS unclear
**Gross 2012 (46)**	no factors related to RTW	no factors related to RTW
**Gross 2019 (47)**	Wrong patient population	Mean ISS = 13.5 ± 7.2
**Haagsma 2012 (48)**	Wrong outcomes	No RTW as outcome
**Haider 2020 (49)**	Wrong patient population	Median ISS = 10
**Hebert 2000 (50)**	Wrong patient population	Mean ISS 17.27 ± 10.61; Range 4-75
**Hepp 2011 (51)**	Wrong patient population	ISS Range = 10-51
**Herrera-Escobar 2018 (52)**	Wrong patient population	Mean ISS =14
**Herrera-Escobar 2019a (53)**	Wrong patient population	70% of patients with ISS 9-15
**Herrera-Escobar 2019b (54)**	Wrong patient population	Mean ISS 14.3±7.3
**Hodgson 2018 (55)**	Wrong patient population	No traumatological study population
**Holmes 2007 (56)**	Wrong outcomes	No desirable outcome related to RTW investigated
**Horn 2021 (57)**	Wrong patient population	Mean ISS = 5
**Hours 2010 (58)**	Wrong patient population	Mixed study population of minorly, moderately, and severely injured

**Hours 2013 (59)**	Wrong patient population	Mixed study population of minorly, moderately, and severely injured
**Hung 2022 (60)**	Wrong patient population	Median ISS =10
**Iakova 2012 (61)**	Wrong patient population	No polytrauma population
**Ioannou 2018 (62)**	Injury Severity unknown /unclear	ISS unclear
**Kabak 2003 (63)**	Wrong patient population	ISS Range 12-66
**Kellezi 2017 (64)**	Wrong patient population	Only a few patients with serious injuries included
**Kendrick 2012 (65)**	Injury Severity unknown /unclear	Severity of included injuries unclear
**Kendrick 2017 (66)**	Wrong patient population	Only 18% of patients with serious injuries
**Kendrick 2018 (67)**	Wrong patient population	Only about 20% of patients with serious injuries
**Kendrick 2021 (68)**	Wrong patient population	Inclusion criteria: ISS >8
**Kissinger 2008 (69)**	Wrong study design	Literature review
**Kruithof 2020 (70)**	Wrong patient population	Median ISS = 5
**Kulmala 2019 (71)**	Injury Severity unknown /unclear	severe injury was defined as an occupational injury that caused 30 or more days of consecutive sick-leave.
**Lange 2007 (72)**	Wrong patient population	Mean ISS = 12.6
**Larsen 2016 (73)**	Wrong patient population	Mean ISS 14.6 ± 11.4
**Lau 2020 (74)**	Wrong patient population	55.4% of the study population with an ISS <16
**Lehmann 1997 (75)**	Injury Severity unknown /unclear	Study used the Hannover Polytrauma Schlüssel to determine the severity of injuries
**Lehmann 1999 (76)**	no factors related to RTW	no factors related to RTW
**Lilley 2012 (77)**	Injury Severity unknown /unclear	ISS unclear
**Lippert-Gruner 2007 (78)**	no factors related to RTW	No factors analyzed
**MacKenzie 1987 (79)**	Injury Severity unknown /unclear	No ISS reported
**MacKenzie 1988 (80)**	Injury Severity unknown /unclear	No ISS reported
**MacKenzie 1998 (81)**	Wrong patient population	Only 24% of patients with ISS >16
**MacKenzie 2006 (82)**	Injury Severity unknown /unclear	ISS unclear
**Madhu 2007 (83)**	Wrong patient population	ISS range 9-27
**Maher 2015 (84)**	Injury Severity unknown /unclear	ISS unclear
**Marasco 2015 (85)**	Wrong patient population	ISS ><16
**Meerding 2004 (86)**	Injury Severity unknown /unclear	Severity of injury was defined as number of injuries and motor vehicle involvement
**Michaels 1998 (87)**	Wrong patient population	Mean ISS = 14 ± 10
**Morris 1991 (88)**	Injury Severity unknown /unclear	No ISS reported
**Murgatroyd 2016 (89)**	Wrong patient population	Most patients with ISS<16
**National Taiwan University Hospital 2015 (90) → ongoing study**	no factors related to RTW	no RTW as an outcome
**Nehra 2019 (91)**	Wrong patient population	Mean ISS =15
**Nguyen 2017 (92)**	Wrong patient population	Nearly 50% of patients with ISS <16
**Nhac-Vu 2014 (93)**	Injury Severity unknown /unclear	>50% with mild and moderate injuries; range of NISS 0-8
**Nyberg 2003 (94)**	Injury Severity unknown /unclear	ISS unclear
**O'Donnell 2010 (95)**	Wrong patient population	Group 1 mean ISS 12.46<br>Group 2 mean ISS 11.54
**O'Toole 2008 (96)**	Wrong patient population	Only 84 patients of the study population with an ISS >17
**Ott 1996 (97)**	Injury Severity unknown /unclear	Study used the Hannover Polytrauma Schlüssel to determine the severity of injuries
**Padovani 2016 (98)**	no factors related to RTW	No factors related to RTW explored
**Palmer 2020 (99)**	Wrong patient population	57% der population with ISS <16
**Parks 2007 (100)**	Injury Severity unknown /unclear	Injury Severity unknown /unclear
**Pelissier 2017 (101)**	Wrong patient population	Patient population: Patients with one severe injury defined as an injury M-AIS3+
**Pelissier 2020 (102)**	Wrong patient population	Mixed population of mild, moderate, and severe injuries
**Prang 2015 (103)**	Wrong patient population	Study population are no polytrauma patients
**Pransky 2005 (104)**	Injury Severity unknown /unclear	Severity of injury unclear
**Rainer 2018 (105)**	Wrong patient population	ISS >< 16
**Redmill 2006 (106)**	Wrong patient population	Includes children 2-18 years
**Richmond 2003 (107)**	Wrong patient population	Mean ISS 13.46 ± 8.42
**Ringgren 2020 (108)**	Injury Severity unknown /unclear	includes patients who had an ambulance dispatched following a traffic accident – severity of injuries unknown
**Rissanen 2019 (109)**	Wrong patient population	Only 27% with MAIS 3
**Savitsky 2020 (110)**	Wrong patient population	90% of study population with an ISS <16
**Schnyder 2003 (111)**	Wrong patient population	Mean ISS 22±10
**Seekamp 1994 (112)**	Injury Severity unknown /unclear	Study used the Hannover Polytrauma Schlüssel to determine the severity of injuries
**Seekamp 1996 (113)**	no factors related to RTW	Describes no factors related to RTW
**Simmel 2018 (114)**	Wrong study design	Literature review
**Simmel 2021 (115)**	Wrong study design	Literature review
**Soberg 2008 (116)**	Injury Severity unknown /unclear	Study used the NISS to determine the severity of injuries
**Soberg 2011 (117)**	Wrong patient population	NISS & ISS/ for RTW group mean ISS 25.8 ±10.8

**Spreadborough 2018 (118)**	Wrong patient population	45% of the study population with an ISS >16
**Tate 1992 (119)**	Wrong patient population	Reports only on patients with isolated fractures
**Toien 2012 (120)**	Wrong patient population	Mean ISS = 13.7 ± 12.97
**Uleberg 2019 (121)**<br>**→ study protocol: Norwegian University of Science 2015 (122)**	Wrong patient population	Only 16% of patient population with an ISS >16
**Urquhart 2006 (123)**	Wrong patient population	30% of the study population with isolated orthopedic injuries
**Van Delft-Schreurs 2014 (124)**	Wrong outcomes	Investigates health related quality of life
**Van der Sluis 1998 (125)**	Na factors related to RTW	no factors related to RTW
**Van der Vlegel 2021 (126)**	no factors related to RTW	no factors related to RTW
**Visser 2021 (127)**	Wrong patient population	>50% of the patient population with an ISS <16
**Wudel 1991 (128)**	Wrong patient population	Mean ISS between 10 and 35.7 (depending on the group)
**Zeckey 2011 (129)**	no factors related to RTW	no factors related to RTW

References:

1. Abedzadeh-Kalahroudi, Razi, Sehat, Asadi Lari. Measurement of Disability and Its Predictors Among Trauma Patients: A Follow-up Study. 2015 Aug 1 [cited 2022 Nov 9]; Available from: https://doaj.org/article/23102ab7ccfe49f89705e658a92250ac
2. Abedzadeh-Kalahroudi M, Razi E, Sehat M, Asadi-Lari M. Return to work after trauma: A survival analysis. Chin J Traumatol. 2017 Apr;20(2):67–74.
3. Ahmed W, Alwe R, Wade D. One-year functional outcomes following major trauma: experience of a UK level 1 major trauma centre. Clin Rehabil. 2017 Dec 1;31(12):1646–52.
4. M. Airey, S. M. Chell, A. S. Rigby, C. The epidemiology of disability and occupation handicap resulting from major traumatic injury. Disabil Rehabil. 2001 Jan;23(12):509–15.
5. Anders B, Ommen O, Pfaff H, Lungen M, Lefering R, Thum S, et al. Direct, indirect, and intangible costs after severe trauma up to occupational reintegration-An empirical analysis of 113 seriously injured patients. GMS Psycho-Soc-Med. 2013;10(Auerbach, K., Otte, D., Jansch, M., Lefering, R. (2009). Medizinische Folgen von Strasenverkehrsunfallen: Drei Datenquellen, drei Methoden, unterschiedliche Ergebnisse? Bergisch Gladbach: Bundesanstalt fur Strasenwesen; 2009. Available from: http:/).
6. Anke AGW, Stanghelle JK, Finset A, Roaldsen KS, Pillgram-Larsen J, Fugl-Meyer AR. Long-Term Prevalence of Impairments and Disabilities after Multiple Trauma: J Trauma Inj Infect Crit Care. 1997 Jan;42(1):54–61.
7. Athanasou JA. Compensable injury and quality of life. Aust J Rehabil Couns. 2015;21(1):18–28.
8. Bai Z, Song D, Deng H, Li-Tsang C. Predictors for return to work after physical injury in China: A one-year review. WORK- J Prev Assess Rehabil. 2018;60(2):319–27.
9. Ballabeni P, Burrus C, Luthi F, Gobelet C, Dériaz O. The effect of recalled previous work environment on return to work after a rehabilitation program including vocational aspects for trauma patients. [Internet]. 2010. Available from: https://www.livivo.de/doc/M20623164
10. Baldry Currens JA. Evaluation of disability and handicap following injury. Injury. 2000;31(2):99–106.
11. Beck B, Stevenson M, Newstead S, Cameron P, Judson R, Edwards ER, et al. Bicycling crash characteristics: An in-depth crash investigation study. Accid Anal Prev. 2016;96:219–27.
12. Beck B, Ekegren CL, Cameron P, Edwards ER, Bucknill A, Judson R, et al. Predictors of recovery in cyclists hospitalised for orthopaedic trauma following an on-road crash. Accid Anal Prev. 2017;106(Health&Mental Health Treatment&Prevention [3300] Amoros, E., Chiron, M., Thelot, B., et al. (2011). The injury epidemiology of cyclists based on a road trauma registry. BMC Public Health, 11, 1, 653http://dx.doi.org/10.1186/1471-2458-11-653Australian B):341–7.
13. Berger-Estilita J, Granja C, Goncalves H, Dias CC, Aragao I, Costa-Pereira A, et al. A new global health outcome score after trauma (GHOST) for disability, cognitive impairment, and health-related quality of life: Data from a prospective cross-sectional observational study. Brain Inj. 2019;33(7):922–31.
14. Brenneman F, Redelmeier D, Boulanger B, McLellan B, Culhane J. Long-term outcomes in blunt trauma: Who goes back to work? J TRAUMA-Inj Infect Crit CARE. 1997;42(5):778–81.
15. Brenner M, Bochicchio G, Bochicchio K, Ilahi O, Rodriguez E, Henry S, et al. Long-term Impact of Damage Control Laparotomy A Prospective Study. Arch Surg. 2011;146(4):395–9.
16. Castillo-Angeles M, Herrera-Escobar J, Toppo A, Sanchez S, Kaafarani H, Salim A, et al. Patient reported outcomes 6 to 12 months after interpersonal violence: A multicenter cohort study. J TRAUMA ACUTE CARE Surg. 2021;91(2):260–4.
17. Chaboyer W, Lee BO, Wallis M, Gillespie B, Jones C. Illness representations predict health-related quality of life 6 months after hospital discharge in individuals with injury: A predictive survey. J Adv Nurs. 2010;66(12):2743–50.
18. Collie A, Simpson PM, Cameron PA, Ameratunga S, Ponsford J, Lyons RA, et al. Patterns and Predictors of Return to Work After Major Trauma: A Prospective, Population-based Registry Study. Ann Surg. 2019;269(5):972–8.
19. Cunha-Diniz F, Taveira-Gomes T, Teixeira J, Magalhaes T. Trauma outcomes in nonfatal road traffic accidents: a Portuguese medico-legal approach. FORENSIC Sci Res.
20. Czaja A, Rivara F, Wang J, Koepsell T, Nathens A, Jurkovich G, et al. Late Outcomes of Trauma Patients With Infections During Index Hospitalization. J TRAUMA-Inj Infect Crit CARE. 2009;67(4):805–14.
21. Daly SL, Gabbe BJ, Climie RE, Ekegren CL. Association between type 2 diabetes and long-term outcomes in middle-aged and older trauma patients. J Trauma Acute Care Surg. 2022;92(1):185–92.
22. de Munter L, Geraerds A, de Jongh M, van der Vlegel M, Steyerberg E, Haagsma J, et al. Prognostic factors for medical and productivity costs, and return to work after trauma. PLOS ONE. 2020;15(3).
23. Denu Z, Yassin M, Yesuf M, Azale T, Biks G, Gelaye K. Disability scores rate changes and predictors among road traffic injury victims admitted at Gondar specialized comprehensive hospital northwest Ethiopia: A prospective follow-up study. TRAFFIC Inj Prev. 2022;23(1):40–5.
24. Dinh MM, Cornwall K, Bein KJ, Gabbe BJ, Tomes BA, Ivers R. Health status and return to work in trauma patients at 3 and 6 months post-discharge: an Australian major trauma centre study. Eur J Trauma Emerg Surg. 2016;42(4):483–90.
25. Doan H, Hobday M, Leavy J, Jancey J. Functional status, pain and return to work of injured motorcyclists involved in a motorcycle crash over one-year post-injury in Vietnam. Inj-Int J CARE Inj. 2020;51(4):924–9.

26. Duckworth MP, Iezzi T. Motor vehicle collisions and their consequences-Part II: Predictors of impairment and disability. Psychol Inj Law. 2018;11(3):288–306.
27. Faux SG, Kohler F, Mozer R, Klein LA, Courtenay S, d'Amours SK, et al. The ROARI project -- Road Accident Acute Rehabilitation Initiative: a randomised clinical trial of two targeted early interventions for road-related trauma [with consumer summary]. Clin Rehabil 2015 Jul297639-652. 2015;
28. Fleischhacker E, Linhart C, Kammerlander C, Bocker W, Zeckey C, Helfen T. Traumatic injuries of the extremities in the emergency room. Notf RETTUNGSMEDIZIN. 2020;23(6):419–28.
29. Folkard SS, Bloomfield TD, Page PRJ, Wilson D, Ricketts DM, Rogers BA. Factors affecting planned return to work after trauma: A prospective descriptive qualitative and quantitative study. Injury. 2016;47(12):2664–70.
30. Fox N, Crutchfield M, LaChant M, Ross S, Seamon M. Early abdominal closure improves long-term outcomes after damage-control laparotomy. J TRAUMA ACUTE CARE Surg. 2013;75(5):854–8.
31. Gabbe BJ, Cameron PA, Hannaford AP, Sutherland AM, McNeil JJ. Routine follow up of major trauma patients from trauma registries: What are the outcomes? J Trauma. 2006;61(6):1393–9.
32. Gabbe B, Cameron P, Williamson O, Edwards E, Graves S, Richardson M. The relationship between compensable status and long-term patient outcomes following orthopedic trauma. Med J Aust. 2007;187(1):14–7.
33. Gabbe BJ, Simpson PM, Sutherland AM, Wolfe R, Lyons RA, Cameron PA. Evaluating time points for measuring recovery after major trauma in adults. Ann Surg. 2013;257(1):166–72.
34. Gabbe BJ, Hofstee DJ, Esser M, Bucknill A, Russ MK, Cameron PA, et al. Functional and return to work outcomes following major trauma involving severe pelvic ring fracture. ANZ J Surg. 2015;85(10):749–54.
35. Gabbe BJ, Simpson PM, Cameron PA, Ekegren CL, Edwards ER, Page R, et al. Association between perception of fault for the crash and function, return to work and health status 1 year after road traffic injury: a registry-based cohort study. BMJ Open. 2015;5(11):e009907.
36. Gabbe BJ, Simpson PM, Harrison JE, Lyons RA, Ameratunga S, Ponsford J, et al. Return to Work and Functional Outcomes After Major Trauma: Who Recovers, When, and How Well? Ann Surg. 2016;263(4):623–32.
37. Gabbe B, Stewart I, Veitch W, Beck B, Cameron P, Russ M, et al. Long-term outcomes of major trauma with unstable open pelvic fractures: A population-based cohort study. TRAUMA-Engl. 2021;23(2):111–9.
38. Gabbe BJ, Braaf S, Cameron PA, Berecki-Gisolf J. Epidemiology and 6- and 12-Month Outcomes of Intimate Partner Violence and Other Violence-Related Traumatic Brain Injury in Major Trauma: A Population-Based Trauma Registry Study. J Head Trauma Rehabil. 2022;37(1):E1–9.
39. Giummarra MJ, Simpson P, Gabbe BJ. Pain, anxiety, and depression in the first two years following transport-related major trauma: A population-based, prospective registry cohort study. Pain Med. 2020;21(2):291–307.
40. Giummarra M, Murgatroyd D, Tran Y, Adie S, Mittal R, Ponsford J, et al. Health and return to work in the first two years following road traffic injury: a comparison of outcomes between compensation claimants in Victoria and New South Wales, Australia. Inj-Int J CARE Inj. 2020;51(10):2199–208.
41. Giummarra M, Xu R, Guo Y, Dipnall J, Ponsford J, Cameron P, et al. Driver, Collision and Meteorological Characteristics of Motor Vehicle Collisions among Road Trauma Survivors. Int J Environ Res Public Health. 2021;18(21).
42. GLANCY K, GLANCY C, LUCKE J, MAHURIN K, RHODES M, TINKOFF G. A STUDY OF RECOVERY IN TRAUMA PATIENTS. J TRAUMA-Inj Infect Crit CARE. 1992;33(4):602–9.
43. Graham C, Yuen K, Yeung J, Poon W, Ho H, Kam C, et al. Predicting probability of return to work at four years after moderate and major trauma in Hong Kong: prospective, multi-center, cohort study. 2016;68(4 Supplement 1):S145.
44. Gray SE, Hassani-Mahmooei B, Kendall E, Cameron ID, Kenardy J, Collie A. Factors associated with graduated return to work following injury in a road traffic crash. J Transp Health. 2018;10(Occupational Interests&Guidance [3610] Adams, H., Ellis, T., Stanish, W.D., Sullivan, M.J. (2007). Psychosocial factors related to return to work following rehabilitation of whiplash injuries. J. Occup. Rehabil., 17, 305-315. http://dx.doi.org/10.1007/):167–77.
45. Gray S, Collie A. Work absence following road traffic crash in Victoria, Australia: A population-based study. Inj-Int J CARE Inj. 2019;50(7):1293–9.
46. Gross T, Schüepp M, Attenberger C, Pargger H, Amsler F. Outcome in polytraumatized patients with and without brain injury. Acta Anaesthesiol Scand. 2012 Oct;56(9):1163–74.
47. Gross T, Morell S, Amsler F. Gender-Specific Improvements in Outcome 1 and 2 Years After Major Trauma. J Surg Res. 2019;235:459–69.
48. Haagsma JA, Ringburg AN, van Lieshout EMM, van Beeck EF, Patka P, Schipper IB, et al. Prevalence rate, predictors and long-term course of probable posttraumatic stress disorder after major trauma: A prospective cohort study. BMC Psychiatry. 2012;12(Baker, S. P., O'Neill, B., Haddon, W., Jr.., Long, W. B. (1974). The injury severity score: a method for describing patients with multiple injuries and evaluating emergency care. J Trauma 1974, 14(3):187-196.Bisson, J. I., Shepherd, J. P., Joy, D., Prob).
49. Haider AH, Herrera-Escobar JP, Al Rafai SS, Harlow AF, Apoj M, Nehra D, et al. Factors Associated With Long-term Outcomes After Injury: Results of the Functional Outcomes and Recovery After Trauma Emergencies (FORTE) Multicenter Cohort Study. Ann Surg. 2020;271(6):1165–73.
50. Hebert JS, Burnham RS. The effect of polytrauma in persons with traumatic spine injury. A prospective database of spine fractures. Spine Phila Pa 1976. 2000;25(1):55–60.
51. Hepp U, Moergeli H, Buchi S, Bruchhaus-Steinert H, Sensky T, Schnyder U. The long-term prediction of return to work following serious accidental injuries: A follow up study. BMC Psychiatry. 2011;11(Organizational Behavior [3660] Baker, S. P., O'Neill, B. (1976). The injury severity score: an update. The Journal of trauma 1976, 16(11):882-885.Brewin, C. R., Robson, M. J., Shapiro, D. A. (1983). Social and psychological determinants of recovery fro).
52. Herrera-Escobar J, Apoj M, Weed C, Harlow A, Al Rafai S, Lilley E, et al. Association of pain after trauma with long-term functional and mental health outcomes. J TRAUMA ACUTE CARE Surg. 2018;85(4):773–9.
53. Herrera-Escobar J, Rivero R, Apoj M, Geada A, Villanyi M, Blake D, et al. Long-term social dysfunction after trauma: What is the prevalence, risk factors, and associated outcomes? SURGERY. 2019;166(3):392–7.
54. Herrera-Escobar J, Seshadri A, Rivero R, Toppo A, Al Rafai S, Scott J, et al. Lower education and income predict worse long-term outcomes after injury. J TRAUMA ACUTE CARE Surg. 2019;87(1):104–10.
55. Hodgson CL, Haines KJ, Bailey M, Barrett J, Bellomo R, Bucknall T, et al. Predictors of return to work in survivors of critical illness. J Crit Care. 2018;48:21–5.
56. Holmes A, Hodgins G, Adey S, Menzel S, Danne P, Kossmann T, et al. Trial of interpersonal counselling after major physical trauma. Aust N Z J Psychiatry. 2007;41(11):926–33.

57. Horn L, de Munter L, Papageorgiou G, Lansink K, de Jongh M, Joosen M. Association of longitudinal changes in patient-reported health status with return to work in the first 2 years after traumatic injury: a prospective cohort study in the Netherlands. BMJ OPEN. 2021;11(12).
58. Hours M, Bernard M, Charnay P, Chossegros L, Javouhey E, Fort E, et al. Functional outcome after road-crash injury: Description of the ESPARR victims cohort and 6-month follow-up results. Accid Anal Prev. 2010;42(2):412–21.
59. Hours M, Chossegros L, Charnay P, Tardy H, Nhac-Vu HT, Boisson D, et al. Outcomes one year after a road accident: Results from the ESPARR cohort. Accid Anal Prev. 2013;50(AAAM, 1990. The Abbreviated Injury Scale, 1990 revision. Des Plaines, IL 60018, USA, p. 74. Amoros, E., Martin, J.-L., Laumon, B. (2006). Under-reporting of road crash casualties in France. Accident Analysis and Prevention, 38, 627-635. http://dx.doi.org):92–102.
60. Hung YP, Bredella MA, Lobmaier IVK, Lozano-Calderón SA, Rosenberg AE, Nielsen GP. Aneurysmal bone cyst and osteoblastoma after neoadjuvant denosumab: histologic spectrum and potential diagnostic pitfalls. APMIS Acta Pathol Microbiol Immunol Scand. 2022;130(4):206–14.
61. Iakova M, Ballabeni P, Erhart P, Seichert N, Luthi F, Deriaz O. Self Perceptions as Predictors for Return to Work 2 Years After Rehabilitation in Orthopedic Trauma Inpatients. J Occup Rehabil. 2012;22(4):532–40.
62. Ioannou L, Cameron PA, Gibson SJ, Ponsford J, Jennings PA, Georgiou-Karistianis N, et al. Financial and recovery worry one year after traumatic injury: A prognostic, registry-based cohort study. Injury. 2018;49(5):990–1000.
63. Kabak S, Halici M, Tuncel M, Avsarogullari L, Baktir A, Basturk M. Functional outcome of open reduction and internal fixation for completely unstable pelvic ring fractures (Type C) - A report of 40 cases. J Orthop TRAUMA. 2003;17(8):555–62.
64. Kellezi B, Coupland C, Morriss R, Beckett K, Joseph S, Barnes J, et al. The impact of psychological factors on recovery from injury: A multicentre cohort study. Soc Psychiatry Psychiatr Epidemiol Int J Res Soc Genet Epidemiol Ment Health Serv. 2017;52(7):855–66.
65. Kendrick Denise, Vinogradova Yana, Coupland Carol, Christie Nicola, Lyons Ronan A, Towner Elizabeth L. Getting back to work after injury: the UK Burden of Injury multicentre longitudinal study. 2012 Aug 1 [cited 2022 Nov 9]; Available from: https://doi.org/10.1186/1471-2458-12-584
66. Kendrick D, Dhiman P, Kellezi B, Coupland C, Whitehead J, Beckett K, et al. Psychological morbidity and return to work after injury: multicentre cohort study. Br J Gen Pract. 2017;67(661):E555–64.
67. Kendrick D, Baker R, Hill T, Beckett K, Coupland C, Kellezi B, et al. Early risk factors for depression, anxiety and post-traumatic distress after hospital admission for unintentional injury: Multicentre cohort study. J Psychosom Res. 2018;112(Association of the Advancement of Automotive Medicine. (2008). Abbreviated Injury Scale (AIS) 2005-Update 2008 Manuals. Barrington: AAAM.Bjelland, I., et al. (2002). The validity of the hospital anxiety and depression scale: an updated literature review):15–24.
68. Kendrick D, das Nair R, Kellezi B, Morriss R, Kettlewell J, Holmes J, et al. Vocational rehabilitation to enhance return to work after trauma (ROWTATE): protocol for a non-randomised single-arm mixed-methods feasibility study. Pilot Feasibility Stud. 2021;7(1):29.
69. Kissinger DB. Traumatic brain injury and employment outcomes: Integration of the working alliance model. Work J Prev Assess Rehabil. 2008;31(3):309–17.
70. Kruithof N, Polinder S, de Munter L, van de Ree CLP, Lansink KWW, de Jongh MAC. Health status and psychological outcomes after trauma: A prospective multicenter cohort study. PLoS ONE. 2020;15(4).
71. Kulmala J, Luoma A, Koskinen L. Able or unable to work? Life trajectory after severe occupational injury. Disabil Rehabil. 2019;41(18):2192–8.
72. Lange C, Burgmer M, Braunheim M, Heuft G. Prospective analysis of factors associated with work reentry in patients with accident-related injuries. J Occup Rehabil. 2007;17(1):1–10.
73. Larsen P, Goethgen CB, Rasmussen S, Iyer AB, Elsoe R. One-year development of QOL following orthopaedic polytrauma: a prospective observational cohort study of 53 patients. Arch Orthop Trauma Surg. 2016 Nov;136(11):1539–46.
74. Lau G, Gabbe BJ, Collie A, Ponsford J, Ameratunga S, Cameron PA, et al. The association between fault attribution and work participation after road traffic injury: A registry-based observational study. J Occup Rehabil. 2020;30(2):235–54.
75. Lehmann U, Gobiet W, Regel G, AlDhaher S, Khah B, Steinbeck K, et al. Functional, neuropsychological and social outcome of multiple trauma patients with severe head injury. UNFALLCHIRURG. 1997;100(7):552–60.
76. Lehmann U, Pape HC, Seekamp A, Gobiet W, Zech S, Winny M, et al. Long Term Results after Multiple Injuries Including Severe Head Injury. Eur J Surg. 1999 Dec 31;165(12):1116–20.
77. Lilley R, Davie G, Ameratunga S, Derrett S. Factors predicting work status 3 months after injury: results from the Prospective Outcomes of Injury Study. BMJ OPEN. 2012;2(2).
78. Lippert-Gruner M, Maegele M, Haverkamp H, Klug N, Wedekind C. Health-related quality of life during the first year after severe brain trauma with and without polytrauma. Brain Inj. 2007;21(5):451–5.
79. MacKenzie EJ, Shapiro S, Smith RT, Siegel JH, Moody M, Pitt A. Factors influencing return to work following hospitalization for traumatic injury. Am J Public Health. 1987 Mar;77(3):329–34.
80. MacKENZIE EJ, Siegel JH, Shapiro S, Moody M, Smith RT. Functional Recovery and Medical Costs of Trauma: An Analysis by Type and Severity of Injury: J Trauma Inj Infect Crit Care. 1988 Mar;28(3):281–97.
81. MacKenzie EJ, Morris JA, Jurkovich GJ, Yasui Y, Cushing BM, Burgess AR, et al. Return to work following injury: the role of economic, social, and job-related factors. Am J Public Health. 1998 Nov;88(11):1630–7.
82. MacKenzie E, Bosse MJ, Kellam J, et al. Early predictors of long-term work disability after major limb trauma. J Trauma. 2006;(61):688–94.
83. Madhu TS, Raman R, Giannoudis PV. Long-term outcome in patients with combined spinal and pelvic fractures. Injury. 2007;38(5):598–606.
84. Maher J, Lindsay J, Tanner C. Mothers caring through injury: How can we understand the dual burden of caregivers' recovery? J Fam Stud. 2015;21(1):72–86.
85. Marasco S, Lee G, Summerhayes R, Fitzgerald M, Bailey M. Quality of life after major trauma with multiple rib fractures. Injury. 2015 Jan;46(1):61–5.
86. Meerding WJ, Looman CWN, Essink-Bot ML, Toet H, Mulder S, van Beeck EF. Distribution and determinants of health and work status in a comprehensive population of injury patients. J Trauma. 2004 Jan;56(1):150–61.
87. Michaels AJ, Michaels CE, Moon CH, Zimmerman MA, Peterson C, Rodriguez JL. Psychosocial factors limit outcomes after trauma. J Trauma. 1998;44(4):644–8.
88. Morris JA, Sanchez AA, Bass SM, MacKenzie EJ. Trauma patients return to productivity. J Trauma. 1991 Jun;31(6):827–33; discussion 833-4.
89. Murgatroyd DF, Harris IA, Tran Y, Cameron ID, Murgatroyd D. Predictors of return to work following motor vehicle related orthopaedic trauma. BMC Musculoskelet Disord. 2016;17:171.

90. National Taiwan University Hospital. Long-term Outcome Related Prognostic Factor and Biomarkers of Major Trauma Database Analysis. 2015;
91. Nehra D, Herrera-Escobar J, Al Rafai S, Havens J, Askari R, Nitzschke S, et al. Resilience and long-term outcomes after trauma: An opportunity for early intervention? J TRAUMA ACUTE CARE Surg. 2019;87(4):782–7.
92. Nguyen TQ, Simpson PM, Braaf SC, Gabbe BJ. Mortality, functional and return to work outcomes of major trauma patients injured from deliberate self-harm. Injury. 2017;48(1):184–94.
93. Nhac-Vu H, Hours M, Chossegros L, Charnay P, Tardy H, Martin J, et al. Prognosis of Outcome in Adult Survivors of Road Accidents in France: One-Year Follow-Up in the ESPARR Cohort. TRAFFIC Inj Prev. 2014;15(2):138–47.
94. Nyberg E, Stieglitz RD, Frommberger U, Berger M. [Psychological disorders after severe occupational accidents]. Versicherungsmedizin. 2003;55(2):76–81.
95. O'Donnell M, Creamer M, McFarlane A, Silove D, Bryant R. Does access to compensation have an impact on recovery outcomes after injury? Med J Aust. 2010;192(6):328–33.
96. O'Toole RV, Castillo RC, Pollak AN, Mackenzie EJ, Bosse MJ. Surgeons and their patients disagree regarding cosmetic and overall outcomes after surgery for high-energy lower extremity trauma. [Internet]. 2009. Available from: https://www.livivo.de/doc/M19858980
97. Ott R, Holzer U, Spitzenpfeil E, Kastl S, Rupprecht H, Hennig F. Quality of life after severe multiple trauma. UNFALLCHIRURG. 1996;99(4):267–74.
98. Padovani C, Da Silva JM, Rotta BP, Neto Rde C, Fu C, Tanaka C. Recovery of functional capacity in severe trauma victims at one year after injury: association with trauma-related and hospital stay aspects. J Phys Ther Sci. 2016;28(5):1432–7.
99. Palmer CS, Cameron PA, Gabbe BJ. Comparison of revised Functional Capacity Index scores with Abbreviated Injury Scale 2008 scores in predicting 12-month severe trauma outcomes. Inj Prev. 2020;26(2):138–46.
100. Parks JK, Diaz-Arrastia R, Gentilello LM, Shafi S. Postinjury Employment as a Surrogate for Functional Outcomes: A Quality Indicator for Trauma Systems. Bayl Univ Med Cent Proc. 2010 Oct;23(4):355–8.
101. Pelissier C, Fort E, Fontana L, Charbotel B, Hours M. Factors associated with non-return to work in the severely injured victims 3 years after a road accident: A prospective study. Accid Anal Prev. 2017;106(Ahman, Sofia, Stalnacke, Britt-Marie. (2008). Post-traumatic stress, depression, and anxiety in patients with injury-related chronic pain: a pilot study. Neuropsychiatr. Dis. Treat. 4(6), 1245-1249.193374652009-02991-019Anke, A.G., Stanghelle, J.K., Fin):411–9.
102. Pelissier C, Fort E, Fontana L, Hours M. Medical and socio-occupational predictive factors of psychological distress 5 years after a road accident: A prospective study. Soc Psychiatry Psychiatr Epidemiol Int J Res Soc Genet Epidemiol Ment Health Serv. 2020;55(3):371–83.
103. Prang K, Berecki-Gisolf J, Newnam S. Recovery from musculoskeletal injury: the role of social support following a transport accident. Health Qual LIFE OUTCOMES. 2015;13.
104. Pransky G, Benjamin K, Savageau J, Currivan D, Fletcher K. Outcomes in work-related injuries: A comparison of older and younger workers. Am J Ind Med. 2005;47(2):104–12.
105. Rainer TH, Graham CA, Yeung HH, Poon WS, Ho HF, Kam CW, et al. Assessment of long-term functional outcome in patients who sustained moderate or major trauma: a 4-year prospective cohort study. Hong Kong Med J. 2018;24 Suppl 2(1):30–3.
106. Redmill DA, McIlwee A, McNicholl B, Templeton C. Long term outcomes 12 years after major trauma. Injury. 2006;37(3):243–6.
107. Richmond TS, Kauder D, Hinkle J, Shults J. Early predictors of long-term disability after injury. Am J Crit Care. 2003;12(3):197–205.
108. Ringgren K, Mills E, Christensen E, Mortensen R, Torp-Pedersen C, Kragholm K. Mortality and return to work in patients transported by emergency ambulance after involvement in a traffic accident. BMC Emerg Med. 2020;20(1).
109. Rissanen R, Liang Y, Moeller J, Nevriana A, Berg H, Hasselberg M. Trajectories of sickness absence after road traffic injury: a Swedish register-based cohort study. BMJ OPEN. 2019;9(7).
110. Savitsky B, Radomislensky I, Goldman S, Gitelson N, Frid Z, Peleg K. Returning to Work Following an Injury: Practical Usage of a Predictive Model Based on a Nationwide Study. J COMMUNITY Health. 2020;45(1):183–93.
111. Schnyder U, Moergeli H, Klaghofer R, Sensky T, Buchi S. Does Patient Cognition Predict Time Off From Work After Life-Threatening Accidents? Am J Psychiatry. 2003 Nov;160(11):2025–31.
112. Seekamp A, Regel G, Bauch S, Takacs J, Tscherne H. [Long-term results of therapy of polytrauma patients with special reference to serial fractures of the lower extremity]. Unfallchirurg. 1994;97(2):57–63.
113. Seekamp A, Regel G, Tscherne H. Rehabilitation and reintegration of multiply injured patients: an outcome study with special reference to multiple lower limb fractures. Injury. 1996;27(2):133–8.
114. Simmel S. [Rehabilitation after Multiple Trauma]. Rehabil Stuttg. 2018;57(2):127–37.
115. Simmel S. [Rehabilitation after multiple traumata]. Orthopade. 2021;50(11):910–5.
116. Soberg H, Finset A, Roise O, Bautz-Holter E. Identification and comparison of rehabilitation goals after multiple injuries: An ICF analysis of the patients', physiotherapists' and other allied professionals' reported goals. J Rehabil Med. 2008;40(5):340–6.
117. Soberg HL. Returning to work after severe multiple injuries: multidimensional functioning and the trajectory from injury to work at 5 years. J Trauma. 2011;71(2):425–34.
118. Spreadborough S, Radford K, das Nair R, Brooks A, Duffy M. A study of outcomes of patients treated at a UK major trauma centre for moderate or severe injuries one to three years after injury. Clin Rehabil. 2018;32(3):410–8.
119. TATE D. WORKERS DISABILITY AND RETURN TO WORK. Am J Phys Med Rehabil. 1992;71(2):92–6.
120. Toien K, Skogstad L, Ekeberg O, Myhren H, Bredal I. Prevalence and predictors of return to work in hospitalised trauma patients during the first year after discharge: A prospective cohort study. Inj-Int J CARE Inj. 2012;43(9):1606–13.
121. Uleberg O, Pape K, Kristiansen T, Romundstad PR, Klepstad P. Population-based analysis of the impact of trauma on longer-term functional outcomes. Br J Surg. 2019;106(1):65–73.
122. Norwegian University of Science, Technology, St. Olavs Hospital, Statistics Norway, Namsos Hospital, Helse Nord-Trøndelag HF, et al. Return to Work After Potential Severe Injury. 2015;
123. VOTOR Project Grp, Urquhart D, Williamson O, Gabbe B, Cicuttini F, Cameron P, et al. Outcomes of patients with orthopaedic trauma admitted to level 1 trauma centres. ANZ J Surg. 2006;76(7):600–6.
124. van Delft-Schreurs CCHM, van Bergen JJM, de Jongh M a. C, van de Sande P, Verhofstad MHJ, de Vries J. Quality of life in severely injured patients depends on psychosocial factors rather than on severity or type of injury. Injury. 2014 Jan;45(1):320–6.
125. van der Sluis CK. Long-term physical, psychological and social consequences of severe injuries. Injury. 1998;29(4):281–5.
126. van der Vlegel M, Haagsma JA, Havermans RJM, de Munter L, de Jongh MAC, Polinder S. Long-term medical and productivity costs of severe trauma: Results from a prospective cohort study. PLoS One. 2021;16(6):e0252673.

127. Visser E, Den Oudsten BL, Traa MJ, Gosens T, De Vries J. Patients' experiences and wellbeing after injury: A focus group study. PLoS ONE. 2021;16(1).
128. WUDEL J, MORRIS J, YATES K, WILSON A, BASS S. MASSIVE TRANSFUSION - OUTCOME IN BLUNT TRAUMA PATIENTS. J TRAUMA-Inj Infect Crit CARE. 1991;31(1):1–7.
129. Zeckey C, Hildebrand F, Pape HC, Mommsen P, Panzica M, Zelle BA, et al. Head injury in polytrauma-Is there an effect on outcome more than 10 years after the injury?? Brain Inj. 2011;25(6):551–9.
