# Supplementary material for: Return to work after major trauma: a systematic review
Source: Scand J Trauma Resusc Emerg Med. 2025 Mar 17;33:44. doi: 10.1186/s13049-025-01351-0 (PMC11917110; doi:10.1186/s13049-025-01351-0)
Supplement: Supplementary file 3 — Additional file 3. [file 13049_2025_1351_MOESM3_ESM.pdf]

Additional file 3

Overview return to work including definitions, measurement time points and RTW rates

Study ID	Outcome name	Definition	Sample size	Measurement timepoint											
				6 months			1 year			2 years			<3 years		
				RTW	Partial	Full	RTW	Partial	Full	RTW	Partial	Full	RTW	Partial	Full
Gabbe 2008 (45)	Return to work	Return to work/study (Yes/No) of those previously working	103	58.6%											
Gross 2010 (31)	Capacity to work	“2 years post-injury reduced capacity to work versus non-reduced capacity to work.”<br>– compared to capacity prior to injury<br>– verification via insurance and medical data<br>the authors rated it reduced, when less than 100% or less than prior to injury	115							47%	53%				
Grotz 1997 (32)	Return to work	“Patients were asked about their job situation before and after the accident and their pension status was documented.”	42										64%		
Haas 2021 (34)	Working	“Working in the third calendar year after injury, as indicated by nonzero Y+3 earnings and (2) the change in total annual earnings attributable to the traumatic injury from Y-1 to Y+3”	5,167										79.3%		
Holtslag 2007 (12)	Return to work	“Complete RTW or nearly complete RTW (>80% part-time work) to former employment”	214				79,9%*	21.5%	58.4%						
Kivioja 1990 (36)	Working ability	Working ability before and after the trauma	92										56.5%		
Livingston 2009 (37)	Return to work / school	No definition provided	76										49%		
Post 2006 (38)	Return to work	“Return to work of those in paid labour before injury”	40				87%	14%	71%						
Simmel 2019 (39)	Return to work	“RTW is defined as the general return to gainful employment. Persons who were able to work at the time of the survey were included in the analyses as patients with RTW and persons who were not able to work at the time of the follow-up survey or who were retired due to an accident were included as patients	84										58%		

		without RTW. No differentiation was made as to the extent to which earning capacity was present or which occupational rehabilitation measures were required in the field.”													
Soberg 2007 (40)	Return to work /education	Return to work/education (RTW) was dichotomized into two categories based on patient information: complete RTW, and not complete return to work/education (NRTW). RTW time was also based on patient information. In the NRTW group, sick leave, active sick leave, medical or vocational rehabilitation, or disability pension were assessed. (…)”	1 year n=100<br><br>2 years N=97				61%	33%**	28%	80%	37%**	43%			
Van Ditshuizen 2022 (41)	Return to work	- working age 18-65 years<br><br>“How many days and how many hours per week, they had paid work before and 1 year after trauma.”	100				68%	31%	37%						
Vles 2005 (42)	Ability to return to work	- Measuring unable to work and change of work/ daily activity	127				74%								

Legend:

**The remaining 20.1% were unemployed 12 months post-injury.*

*** defined as “active or part- time sick-leave/rehabilitation compensation.”*
