# Supplementary material for: Return to work after major trauma: a systematic review
Source: Scand J Trauma Resusc Emerg Med. 2025 Mar 17;33:44. doi: 10.1186/s13049-025-01351-0 (PMC11917110; doi:10.1186/s13049-025-01351-0)
Supplement: Supplementary file 4 — Additional file 4. [file 13049_2025_1351_MOESM4_ESM.pdf]

Additional File 4

Factors affecting RTW identified in the included studies of non-regression analyses, univariate and multivariate analyses

Study ID	Number of participants in multivariate analysis	% loss to follow up / missing data	Analysis	Multivariate analysis: significant results	Multivariate analysis: Non-significant results	Univariate analysis: significant results	Factors from other analysis (no regression analysis)
Gabbe 2008 (34)	103	3.4%	logistic regression analyses	• Higher discharge **FIM motor scores**: (AOR 1.03, 95% CI: 1.01–1.04)<br>• **Age** 35 to 44 years: (AOR 0.31, 95% CI: 0.13–0.76)<br>• **accident compensation schemes** for work and transported-related injury: (AOR 0.28; 95% CI: 0.11–0.72)		• compensable status<br>• age<br>• Presence of extremity injury<br>• ISS<br>• Discharge from hospital destination<br>• modified FIM locomotion item<br>• FIM motor score<br>• total FIM score<br>• modified FIM total	
Gross 2010 (33)	115	36.1%%	Logistic regression analysis	• **Educational level** (OR: 0.249; 95%CI 0.068-0.916, p=0.036)<br>• **ISS** (OR:1.115; 95%CI 1.020 – 1.220)<br>• **Time in ER** (OR: 0.917, 95%CI 0.862-0.974 p=0.005)<br>• **LEP** (OR: 1.002, 95%CI 1.000 1.004, p= 0.033)<br>Nagelkerke R^2 0.74		• Pain<br>• EQ-5D<br>• SF-36<br>• FIM<br>• MFA<br>• Time to CT<br>• Time to emergency OR<br>• GCS	

				• **NHP** (OR: 1.103, 95%CI: (1.058 1.150), p=0.001)		• ISS<br>• AIS head<br>• RTS<br>• $SapO_2$ 1 day<br>• TRISS<br>• SAPS II mort<br>• Smoking pre-trauma	
Grotz 1997 (39)	42	27.5%	Chi-Quadrat Test and Mann-Whitney U-Test				**Significant:**<br>• Traumatic brain injury (grade)<br>• LOS Rehab<br>• Limited movement Tegner Activity Score<br><br>**Non-significant:**<br>• Age<br>• Sex<br>• Polytrauma Schlüssel<br>• GCS<br>• GOS<br>• ICU stay<br>• Mechanical ventilation<br>• Limited movement (hands, elbow, shoulder, knee, ankle)
Haas 2021 (41)	5,167	Retrospective analysis	Weighted multivariable probit regression	• **ICU admission** mean difference-in-difference change in per- centage employed: with intensive or special care unit, −22.1% [95% CI, −24.4% to −19.8%]<br>• **Severe head injury** presence of a severe head injury (mean difference-in-difference change in percentage employed: yes, −17.9% [95% CI, −19.9% to −15.8%]	• Sex<br>• Marital status<br>• Self-employment		

				• Longer index **LOS hospital** (mean difference-in-difference change in percentage employed: low tercile, −5.6% [95% CI, −7.3% to −3.8%]<br>• **Mechanical ventilation** (mean difference- in-difference change in percentage employed: yes, −26.8% [95% CI, −30.1% to −23.5%]<br>• Low preinjury **income** (mean difference in difference −18.5% [95% CI, −20.8% to −16.2%]<br>• Age			
Holtslag 2007 (12)	214	7%	logistic regression analysis	**At discharge:** Nagelkerke R^2= 0.23<br>• **Age** (OR 1.89; CI 95% 1.03-3.46)<br>• **Spinal cord injury** (OR 4.3; CI 95% 1.07 – 17.2)<br>• **Hospital LOS** <21 (OR 2.65; CI 95% 1.35 – 5.18)<br>• **Discharge home** (OR 0.41; Ci 95% 0.20 – 0.84)<br><br>**At 12-18 months post-injury** Nagelkerke R^2= 0.507<br>• **GARS-ADL** (OR 3.69; CI 95% 1.71 – 7.95)<br>• **HISCwA** (OR 2.63; CI 95% 1.24 – 5.58)<br>• **AMA** (OR 7.51, CI 95% 3.29 – 17.1)	**At discharge:**<br>• ISS<br>• Comorbidity<br>• ICU stay<br>• Injury location – brain<br><br>**At 12-18 months post-injury**<br>• ISS<br>• Comorbidities<br>• Injury location – brain<br>• ICU stay	• Age<br>• Co-morbidity<br>• Injury location – brain<br>• Injury location – spinal cord<br>• ISS<br>• ICU stay<br>• Discharge home<br>• Discharge to rehab center<br>• AMA<br>• GARS-ADL<br>• HISCwA	
Kivioja 1990 (36)	71	15.6%	Logistic regression	• **ISS**<br>• **Age**<br>• **Physical fitness**<br>• **Complaints from lower extremity injury**			
Livingston 2009 (37)	76	28.6%	Kruskal-Wallis and ^{2} tests /Student's *t* and Mann-	/			Non-sign.<br><br>• Presence of any TBI<br>• Head AIS<br>• Extremity AIS

			Whitney *U* tests				• Presence of any extremity fracture<br>• ISS<br>• Age<br>• Ventilator days
Post 2006 (43)	40	24.3%	chi-square tests / Student's t-tests	/			**Significant**<br>• Age<br>**Non-significant**<br>• Head injury<br>• Extremity injury<br>• ISS<br>• Blue collar and white collar
Simmel 2019 (32)	84	40%	Logistic regression	• **Age** (OR 1.088; p<0.05)<br>• **General health status** (OR 0.934; p<0.01)<br>• **LOS ICU** (OR 1.072; p<0.01)<br>Nagelkerkes R^2 = 0,526	• Time between follow-up and accident	• LOS hospital<br>• Cost bearer for treatment<br>• Head injury<br>• Abdomen injury<br>• More support during hospitalization expected	
Soberg 2007 (31)	97	34.2%	Cox regression model	• **Social functioning** (RR 2.72; CI95% 1.04–7.13)<br>• **LOS hospital** (RR 5.06; CI95% 1.28–20.01)<br>• **Education** (RR 4.14; CI95% 1.99–8.61)	• profession<br>• NISS<br>• powerful other locus of health control<br>• physical functioning<br>• sex<br>• age		
Van Ditshuizen 2022 (38)	100	50%	Chi-square test or Fisher's exact	/			**Sign.**

			test was used as applicable				• Better score in all health domains of EQ-5D<br>• Injury Severity<br><br>**Non-sign.**<br>• Gender<br>• Type of injury<br>• Age<br>• Level of education<br>• No of comorbidities<br>• Severity of specific organ injuries 1-year post-injury
Vles 2005 (35)	127	15%	Logistic regression and linear regression	• **ISS** (*p* < 0.001)<br>• **Gender** (*p* < 0.05)<br>• **Injury of one or more extremities** (protective effect – *p*< 0.05)	• Age<br>• Nr. Of body areas with injury<br>• Head injury<br>• Abdominal injury<br>• Thorax injury<br>• Injury remaining body<br>• Isolated head injury<br>• Injury spine / pelvis		

Legend: AIS = Abbreviated Injury Scale; AMA = percentage of permanent impairment according to the fourth American Medical Association guide; CT= computer tomography; EQ-5D = European Quality of Life 5 Dimensions 3 Level Version; ER = emergency room; FIM = functional independency measurement; GARS-ADL = Groningen Activity Restriction Scale-Activities of Daily Living; GCS = Glasgow Coma Scale; GOS = Glasgow Outcome Scale; HISCwA = Head Injury Symptom Checklist, without anxiety; ICU = intensive care unit, ISS= Injury Severity Score; LEP = Mean nurse per day and per patient ratio; LOS = length of stay, MFA = Musculoskeletal functional Assessment NHP = Nottingham Health Profile, NISS = New injury Severity Score; RTS = Revised Trauma Score; $SapO_2$ = arterial hemoglobin saturation by pulse oximetry; SAPS II mort = expected Simplified Acute Physiology Score II mortality; SF-36 = Short Form 36; TRISS = Trauma and Injury Severity Score
